# Supplementary material for: Statistical laws of stick-slip friction at mesoscale
Source: Nat Commun. 2023 Oct 5;14:6221. doi: 10.1038/s41467-023-41850-1 (PMC10556047; doi:10.1038/s41467-023-41850-1)
Supplement: Supplementary file 1 — Supplementary Information [file 41467_2023_41850_MOESM1_ESM.pdf]

Supplementary Information for:
Statistical laws of stick-slip friction at mesoscale

Caishan Yan[1], Hsuan-Yi Chen[2,3], Pik-Yin Lai[2,3] and Penger Tong[1]
[1] Department of Physics, Hong Kong University of Science and Technology, Clear Water Bay, Kowloon, Hong Kong
[2] Department of Physics and Center for Complex Systems,
National Central University, Taoyuan City 320, Taiwan
[3] Physics Division, National Center for Theoretical Sciences, Taipei 10617, Taiwan
(Dated: September 15, 2023)

CONTENTS

I. Supplementary Methods 1
A. Characterization of rough and smooth substrates 1
B. Calibration of lateral force 3
C. Effective lateral spring constant of the scanning probe 4
D. AFM operation 4

II. Supplementary Discussion on Data Analysis 6
A. Identification of slip events 6
B. Determination of interfacial adhesion 7
C. Determination of the friction coefficient μ_c 7
D. Normal load effects on the frictional force statistics 8
1. PDFs of the normalized maximal force f_c 8
2. PDFs of the slip length δx_s 9
3. PDFs of the local force gradient k' 9
E. PDFs of f_c, δx_s and k at the high-load limit 10
F. Scanning speed effects on the frictional force statistics 12
G. Frictional force fluctuations at mesoscale in the small load regime ($N < N_m$) 13

III. Supplementary Discussion on Theoretical Model 13
A. Frictional force and effective spring constant in the stick state 13
B. An under-damped spring-block model for the slip dynamics 15
C. Numerical results of the under-damped spring-block model 17
D. Steady-state velocity time series of the underdamped spring block model 18
E. Numerical results of the over-damped spring-block model 19

Supplementary References 19

I. SUPPLEMENTARY METHODS

A. Characterization of rough and smooth substrates

An ultra-fine silicon carbide sandpaper taped on a flat glass slide is used as the rough substrate for the friction measurement. The sandpaper has an average grain size of 100 nm, as shown by the AFM topographical image in Fig. 1(c) of the main text. Here we provide additional characterizations of the sandpaper surface. Supplementary Fig. 1(a) shows an AFM topographical image of the sandpaper over an area of 50×50 μm^2, from which we find the RMS roughness of the sandpaper surface to be 0.17 μm (over an area of 50×50 μm^2). It is also seen that the roughness of the sandpaper is quite uniform with very few large-sized pits (> 10 μm). This uniform roughness allows the scanning probe to move steadily without being fully trapped during the scan. Supplementary Fig. 1(b) shows the roughness height profile $h(x)$ along the red

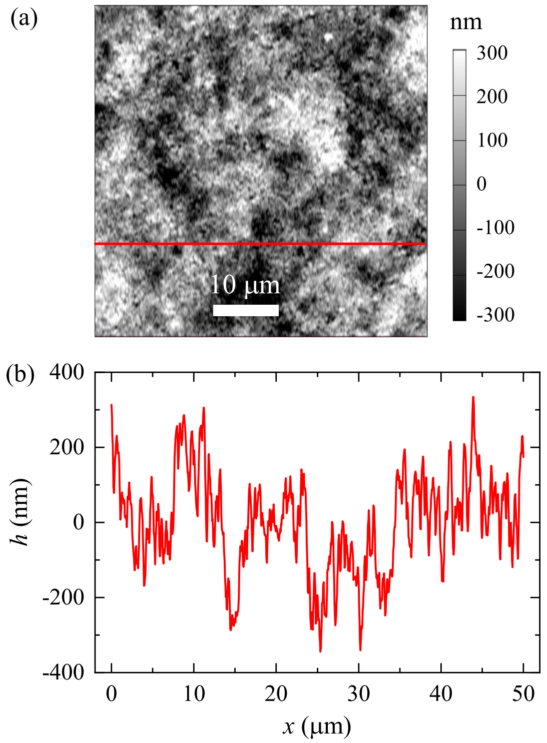

Supplementary Fig. 1. Surface morphology of the sandpaper. (a) An AFM topographical image of the ultra-fine sandpaper surface over an area of 50 μm $\times$ 50 μm. The horizontal scale bar is 10 μm and the vertical grey scale bar shows the surface height of the sandpaper. (b) Variations of the surface roughness height $h(x)$ along the red line marked in (a).

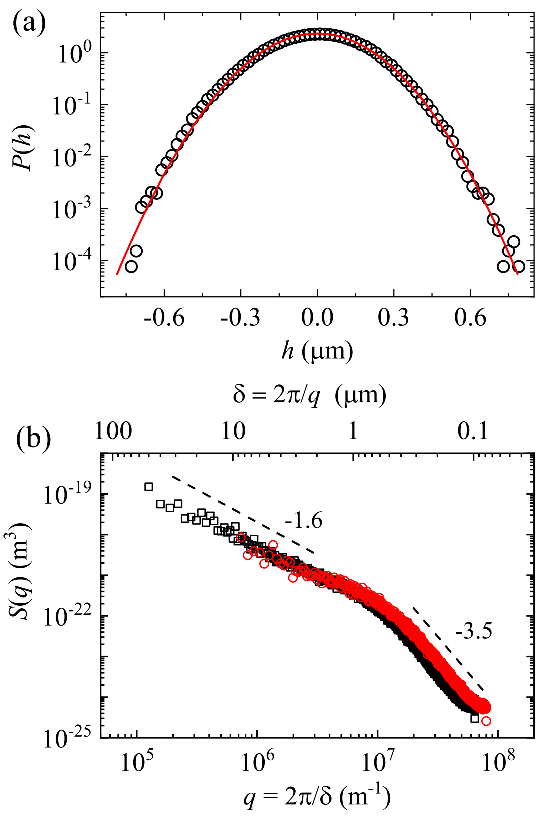

Supplementary Fig. 2. **Characterization of surface roughness of the sandpaper.** (a) Measured PDF $P(h)$ of surface height h. The mean value of h is set to zero. The red line shows a Gaussian fit to the data points with a standard deviation of 0.17 μm. (b) Measured power spectral density (PSD) functions $S(q)$ of the roughness height profile $h(x)$ as a function of the wave-vector $q = 2\pi/\delta$ (bottom axis). The top axis indicates the corresponding wave-length δ. The red circles are obtained from the AFM topographical image shown in Fig. 1(d) of the main text (10×10 μm^2) with a spatial resolution of 0.039 μm. The black squares are obtained from the AFM topographical image shown in Fig. 1(a) (50×50 μm^2) with a spatial resolution of 0.049 μm. The dashed lines indicate the two power-law regions with the numbers indicating the power law exponents.

line marked in Fig. 1(a). The abrupt variations in the obtained $h(x)$ indicate sharp protrusion of the grain clusters on the sandpaper. These grain clusters (or asperities) will exert strong friction (or pinning) to the scanning probe.

We now discuss the statistical properties of the roughness height profile $h(x)$ of the sandpaper. Supplementary Fig. 2(a) shows the probability distribution function (PDF) $P(h)$ of the surface height h, where the mean value of h is set to zero and h is sampled over an area of 50×50 μm^2. It is found that the measured $P(h)$ is well described by a Gaussian distribution with a standard deviation 0.17 μm, which is the RMS roughness.

Supplementary Fig. 2(b) shows the measured power spectral density (PSD) functions $S(q)$ of the roughness height profile $h(x)$. Because the sandpaper surface is isotropic, herein we only consider the one-dimensional PSD. It is seen that the two PSD functions obtained at two different spatial resolutions overlap well in the common range of the wave-vector q. The functional form of the measured $S(q)$ over the entire q-range (10^5 m$^{-1} \lesssim q \lesssim 10^8$ m^{-1}) can be described by two regimes of approximately power-law scaling, $S(q) \sim q^{-\alpha}$, together with a narrow intermediate cross-over region centered around $q \sim 10^7$ m^{-1}. The power-law exponent α in the small-q regime (or long-wavelength regime with $\delta \gtrsim 2$ μm) is $\alpha \simeq 1.6$ (left dashed line) whereas the value of α in the large-q regime (or short-wavelength regime with $\delta \lesssim 0.3$ μm) is $\alpha \simeq 3.5$ (right dashed line). While the cross-over region has a finite width, we find that the two power-law fits to the data meet at a cut-off length $\ell_c \simeq 0.34$ μm. Similar cross-over behaviors from one power-law-like regime to another were also observed for other rough surfaces [1–3].

A power-law-like regime in the measured PSD suggests that the rough surface has some self-similar features and such a surface is referred to as a fractal. By examining the AFM images (Fig. 1(a) and Fig. 1(d) of the main text) of the sandpaper surface, we find that the power-law-like regime at small δ (from ~ 0.1 μm to ~ 0.4 μm) can be attributed to the individual gains (with an average grain size 0.1 μm) and their small clusters (such as dimers, trimers and tetramers). A similar conclusion was also drawn in a previous study [3]. On the other hand, the power-law-like regime at large δ (from $\sim$2 μm to $\sim$30 μm) is caused primarily by the random distribution of larger clusters (aggregation of small clusters), which are associated with the bright regions (or bumps) and dark regions (or depressions) in the AFM topographical images. The cut-off length ℓ_c that separates the two power-law-like regimes, therefore, can be viewed as the lower cutoff of the large cluster (or domain) sizes. Supplementary Fig. 2(b) thus demonstrates that the sandpaper has various asperities (or micro-contacts) with a broad range of sizes.

For our system, the maximum micro-contact size is limited by the dimension of the apparent contact area along the scanning direction, which is ~ 4 μm for the quasi-1D probe and ~ 12 μm for the 2D probe. The minimum micro-contact size, on the other hand, is the size of the grain particles on the sandpaper. Consequently, when the AFM probe scans over the sandpaper, it will experience various asperities (or microscopic contacts) with a broad range of scales from the grain size of ~ 0.1 μm to the probe size up to ~ 12 μm. Within the two length limits, the measured PSD (as a function of wavelength δ) has two (short-ranged) power-law-like regimes, which nevertheless cannot be described by a single fractal.

As mentioned in the main text, the main purpose of using two contact materials with a large difference in hardness is to increase the compliance of the contact so that the number of asperities (or micro-contacts) with different random configurations (e.g., different roughness heights and sizes) is maximized at the contact without

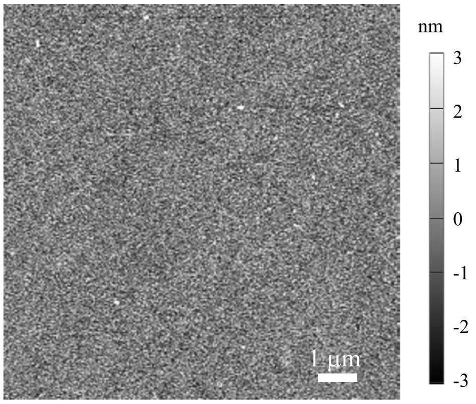

Supplementary Fig. 3. **Surface morphology of the gold-coated silicon wafer.** The AFM topographical image has an dimension of 10 μm$\times$10 μm, where the vertical grey scale indicates the surface height. The RMS roughness of the gold-coated wafer surface is 1.0 nm over the whole scanning area.

introducing large plastic deformations and wear. More specifically, the rigid sandpaper surface used here serves as a base to provide a large number of asperities with a broad range of randomness, whereas the probe surface is made of a softer material and is flat so that it is able to have a close contact with the rough surface of the sandpaper at all length scales and feel the underlying pinning force field, when a small normal load is applied. If the probe surface were made of another rigid sandpaper, for example, the contact between the two hard surfaces is dominated primarily by a small number of the most prominent asperities on each surface. In this case, it would be difficult to have an effective local contact under a regular normal load that can feel many asperities with a broad range of randomness.

To measure the static spring constant k_0 of the scanning probe in contact with a non-slip substrate, we use a smooth silicon wafer surface coated with a thin layer of gold as the non-slip substrate. Supplementary Fig. 3 shows an AFM topographical image of the gold-coated silicon wafer surface, which has a RMS roughness of 1.0 nm over an area of 10 μm$\times$10 μm.

B. Calibration of lateral force

When a lateral (or normal) force is applied to the AFM tip, the cantilever will bend laterally (or vertically) causing a displacement of the laser spot on the photodetector of the AFM. This displacement is recorded by the AFM as a voltage signal proportional to the magnitude of the applied force. To convert this voltage signal to the actual force, one needs to calibrate two proportionality constants. One is the optical sensitivity of the detector, which is the displacement of the tip per voltage change; the other is the spring constant of the probe, which is the force per tip displacement. For the vertical bending mode, the optical sensitivity is extracted from the slope of the voltage-displacement curve measured when the AFM cantilever is pressed against a flat hard surface. The vertical spring constant is obtained using the thermal power spectral density (PSD) method [4, 5]. Using this method, we find the normal spring constant of the AFM cantilevers used in this experiment to be $k_n \simeq 0.7$ N/m.

For the lateral bending mode used in the friction measurement, however, the optical sensitivity usually cannot be obtained using the same method as that for the vertical bending mode because of the undetermined stiffness at the solid interface [6–9]. Furthermore, the thermal PSD method is not applicable to the lateral bending mode. As a result, the lateral spring constant of the cantilever together with the tip is usually estimated based on the sample dimensions.

In this experiment, we make a direct calibration of the lateral force sensitivity, the coefficient that relates the measured voltage signal to the actual lateral force, using a glass fiber as a force sensor. The glass fiber is pulled from a glass rod of 1 mm in diameter using a pipette puller (P-97, Sutter Instrument) and has the dimensions of ~ 10 μm in diameter and ~ 5 mm in length. The fiber is linked to a segment of unstretched glass rod (1 mm in diameter) as a handle for convenience of manipulation. The spring constant of the glass fiber is calibrated by vertically pressing an AFM cantilever of known spring constant (10.9 N/m) against the free end of the horizontally aligned glass fiber, as shown in Supplementary Fig. 4(a). The slope of the measured force-displacement curve is the effective spring constant of the joint cantilever-fiber system, which is composed of two springs connected in series. With the known spring constant of the cantilever, we obtain the spring constant of the glass fiber to be $\simeq 0.029$ N/m, which is very small compared with that of the cantilever.

A similar method is used to calibrate the lateral force sensitivity of the scanning probe. As shown in Supplementary Fig. 4(b), the scanning probe is mounted on the AFM cantilever holder and the glass fiber is mounted horizontally on the AFM scanner with its long axis in parallel with the cantilever arm of the scanning probe. Using the controllers of the AFM cantilever holder and scanner, the glass fiber is moved gradually toward the scanning probe until its tip touches the scanning probe. We then obtain the lateral force-displacement curve, where the measured force is in unit of volt and the displacement is the total deflection of the glass fiber and the scanning probe. Because the spring constant of the glass fiber is much smaller than the lateral spring constant of the scanning probe (~ 1 N/m), the deflection of the scanning probe is negligible. Therefore, by multiplying the displacement by the spring constant of the glass fiber, we convert the force-displacement curve to force (in unit of volt)-force (in unit of Newton) curve and the slope is the proportionality constant between the measured voltage signal

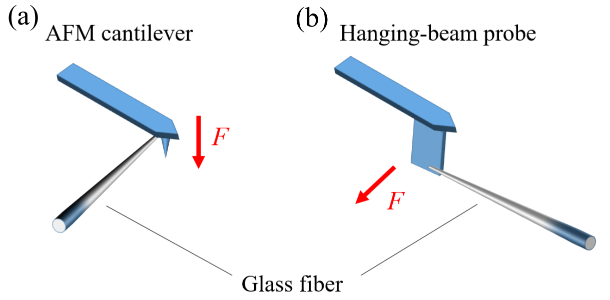

Supplementary Fig. 4. **Calibration of lateral force sensitivity of the scanning probe.** (a) Calibration of a glass fiber, which is deformed under a normal force F applied by an AFM cantilever of known spring constant. (b) Calibration of the scanning probe using the glass fiber as a force sensor. The glass fiber is deformed under a lateral force F applied by the hanging beam probe. The red arrows indicate the direction of the applied forces.

and the actual lateral force.

C. Effective lateral spring constant of the scanning probe

The effective lateral spring constant k_e of the scanning probe as a whole is determined by the contributions from its three components, namely, the horizontal cantilever (k_c), the vertical hanging beam (k_b), and the beam tip with UV cured glue in contact with the substrate (k_t). Because the three springs are connected in series, we have [6]

$$\frac{1}{k_e} = \frac{1}{k_c} + \frac{1}{k_b} + \frac{1}{k_t}. \tag{1}$$

The spring constant of the individual components can be determined by their dimensions and material properties. The lateral spring constant of a rectangular AFM cantilever with a hanging tip is given by [6, 7]

$$k_c = \frac{GWT^3}{3L(\ell + T/2)^2}, \tag{2}$$

where G is the shear modulus of the cantilever ($G \simeq 51$ GPa for silicon [10]), and the cantilever has the dimensions of length L, width W, thickness T and tip length ℓ (or the hanging beam length ℓ). For the probe used in the experiment, we have $L \simeq 300$ μm, $W \simeq 35$ μm, $T \simeq 2$ μm, $\ell \simeq 90$ μm, and the resulting $k_c \simeq 1.9$ N/m.

The lateral spring constant of the hanging beam is actually the normal spring constant when used as an AFM cantilever and is given by [6, 7]

$$k_b = \frac{Ewt^3}{4\ell^3}, \tag{3}$$

where E is the Young's modulus of the hanging beam ($E \simeq 169$ GPa for silicon [10]), and the beam has the dimensions of length ℓ, width w, and thickness t. With $\ell \simeq 90$ μm, $w \simeq 35$ μm, and $t \simeq 2$ μm, we have $k_b \simeq 16$ N/m.

The lateral spring constant of the hanging beam tip in contact with the substrate can be estimated by assuming that the UV cured glue layer and the embedded hanging beam tip functions as a rubber block with an effective shear modulus G_t. In this case, we have

$$k_t = \frac{G_t A_r}{\ell}, \tag{4}$$

where A_r is the real contact area and ℓ is the hanging beam length. When the contact between the hanging beam tip and the substrate is "perfect" such that the real contact area A_r is close to the apparent contact area A_a, k_t reaches its maximal value k_t^{max}. Because the shear modulus of the substrate (either the sandpaper or the Au-coated silicon wafer) is much larger than that of the nanoparticle-embedded UV cured glue, the effective shear modulus G_t should be similar to the shear modulus of the UV cured glue, which is estimated as $G_t = E/[2(1-\nu)] \simeq 3$ MPa (with $E \simeq 3$ MPa and the Poisson's ratio $\nu = 0.5$ for incompressible isotropic materials). Also with $A_r^{max} = A_a \simeq 100$ μm^2 and $\ell \simeq 90$ μm, we have $k_t^{max} \simeq 3.3$ N/m.

With the estimated values of k_c, k_b, and k_t^{max}, we obtain the maximum effective lateral spring constant $k_e^{max} \simeq 1.3$ N/m using Eq. (1). The estimated k_e^{max} is in the same order as the measured static spring constants, $k_0 \simeq 0.66$ N/m for the quasi-1D probe and $k_0 \simeq 0.48$ N/m for the 2D probe. The difference between the two values may result from an overestimation of the real contact area A_r^{max} in Eq. (4) due to the surface roughness of the hanging beam tip with a nanoparticle-glue coating.

D. AFM operation

In the experiment, an AFM (MFP-3D, Asylum Research Inc.) is used for the friction measurement. The AFM operates in the lateral force microscopy mode, in which the scanner drives the substrate (the sandpaper) moving in the direction perpendicular to the cantilever. Before the scan starts, the scanning probe is pressed against the sandpaper under a normal load N. The probe scans on the sandpaper at a constant speed U in one direction for a distance of 100 μm followed by a re-scan along the same track in the reversed direction. During the scanning, the displacement and lateral force signals are recorded at a sampling rate 1 kHz.

In the meso-scale friction experiment, one may examine the level of interfacial wear by checking the reproducibility of the frictional force trajectories $F(x)$ obtained when the scanning probe repeats its scans over the same region of the sandpaper surface and under the same condition. Supplementary Fig. 5 shows three such repeated force trajectories. The three force trajectories overlap with each other in most positions, suggesting that

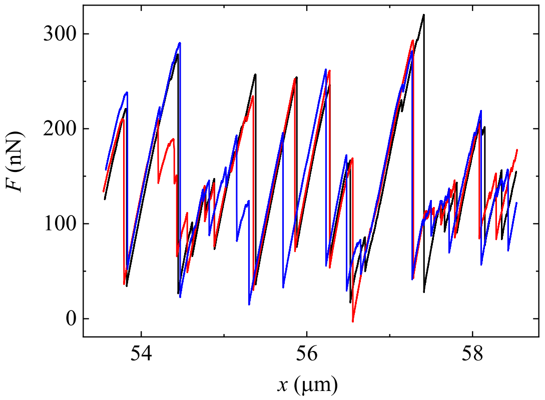

Supplementary Fig. 5. Reproducibility of the frictional force measurements. The black, red and blue curves are obtained sequentially along the same track on the sandpaper under the same scanning conditions: normal load $N = 400$ nN and scanning speed $U = 100$ nm/s. Three scans are made and the total travel distance for each scan is 200 μm.

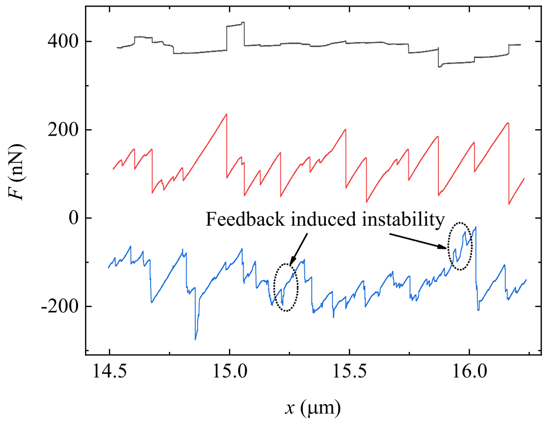

Supplementary Fig. 6. Influence of deflection feedback gain on the frictional force measurements. The two measurements are made at feedback gain values of 0.001 (red curve) and 0.1 (blue curve) under the same normal load $N = 400$ nN (mean value) and same scanning speed $U = 100$ nm/s. For clarity, the origin of the blue curve is shifted downwards by 250 nN. The circled portions of the blue curve show the feedback-induced instabilities. The black curve shows fluctuations of the normal force curve recorded simultaneously with the red curve.

the contact geometry of the interface remains essentially unchanged during the repeated scans and the interfacial wear is not significant. There are a few locations where the three curves show some deviations. These deviations occur at the locations where a large slip is replaced by two or more smaller ones (such as those shown at $x = 54.2$ μm and $x = 55.3$ μm) or small-sized slips take place repeatedly (such as those shown at $x = 54.8$ μm and $x = 57.6$ μm). The deviations may be caused by the stochastic slip dynamics or by some minor plastic deformation of small asperities. With these results, we conclude that by ignoring the plastic deformation of small asperities (if any), the interfacial wear in our system is negligible. To obtain the probability density function with enough independent slip events, we repeat the force measurements five times along different tracks on the sandpaper under the same scanning condition. A total of $\sim$2500 slip events are collected over a total scanning distance of 500 μm under each scanning condition.

A common issue associated with the frictional force measurement using AFM is that the normal load N and the lateral force F are partially coupled because the torsional bending of the cantilever involves both the vertical and lateral components. Therefore, fluctuations of the lateral force F will result in fluctuations of the normal load N. To keep N constant, the deflection feedback control (for normal load) is usually used, which is very helpful for measurement of the frictional force over a flat surface where fluctuations of the frictional force are small. However, the feedback will introduce artificial discontinuities to the measured frictional force over rough surfaces. Suppression of the deflection feedback is, therefore, needed for the AFM study of stick-slip friction. In our experiment, we set the gain of the deflection feedback to a small value ($\sim$0.001) so that the feedback is close to an off state. This is equivalent to set the lateral scan at a constant "indentation". This setting has an advantage over that of completely turning off the feedback control in that the normal load can still be tuned slowly in case there is an incline on the substrate.

Supplementary Fig. 6 shows how the deflection feedback gain affects the measured frictional force F. When the gain is set at 0.001 (red curve), the measured frictional force reveals a clear zigzag pattern for the stick-slip motion of the hanging bean probe. When the gain is set at 0.1 (blue curve), however, the measured F shows many irregular discontinuities, as indicated in Supplementary Fig. 6. These irregular spikes occur when the probe is at the stick state as a result of the fast change of the normal load. When the scanning probe slides against the sandpaper, the normal load signal fluctuates but its fluctuation amplitude is much smaller than its mean value when the gain is set at 0.001. This is shown by the black curve in Supplementary Fig. 6, confirming that the scanning is made under the condition that the normal load is kept approximately constant.

In the experiment, we choose the normal load N in the range of 200–600 nN so that the scanning probe is under a continuing stick-slip motion with irregular stick (and slip) periods. The scanning distance over the sandpaper is set at 100 μm for each scan so that one can collect an adequate amount of slip events to build up the probability density functions. The scanning speed U is kept in the low-speed regime so that individual slips can be fully resolved without any influence from other dynamic effects. With the chosen scanning speed $U = 0.1$ μm/s, each scan takes 16.7 minutes and collects 1×10^6 data points for

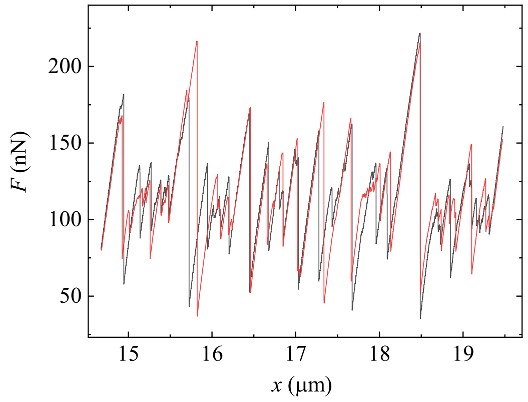

Supplementary Fig. 7. Frictional force measurements at different scanning speeds. The two measurements are made at a scanning speed $U = 0.1$ μm/s (black curve) and $U = 1$ μm/s (red curve) when the 2D scanning probe slides over the same region on the sandpaper and under the same normal load $N = 600$ nN.

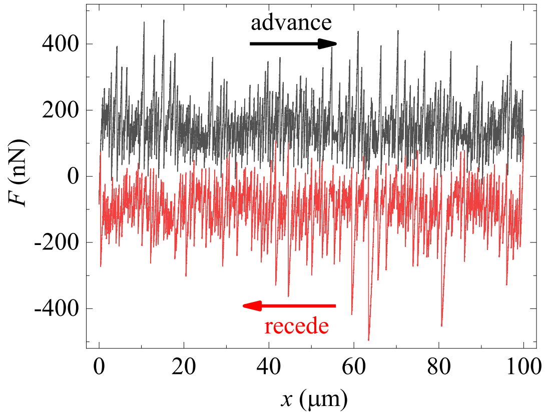

Supplementary Fig. 8. An example of the measured frictional force loop. The measurement is made when the 2D probe is pulled back and forth for a cycle under the normal load $N = 600$ nN and at scanning speed $U = 100$ nm/s.

the measured force trajectory $F(x)$ (at the sampling rate 1 kHz). This is about the slowest scanning speed experimentally accessible for a mechanically stable AFM scan without much influence from noises resulting from mechanical instabilities and drifts of the system. We also vary the scanning speed U in the range 0.1–1 μm/s to verify the reproducibility of the experimental findings.

With our AFM setup, the response time t_0 of the scanning probe is $t_0 \simeq \sqrt{m/k_0}$, where m is the mass and k_0 is the static spring constant of the scanning probe. With $m \simeq 1 \times 10^{-8}$ g estimated based on the dimension of the hanging beam and $k_0 \simeq 0.48$ N/m as measured for the 2D probe, we have $t_0 \simeq 5 \times 10^{-6}$ s. For a typical slip distance $\lambda_0 = 0.42$ μm (see Supplementary Fig. 2(b)), we find a typical slip velocity $\lambda_0/t_0 \simeq 8.4$ cm/s. Because the scanning speeds U used in the experiment are much smaller than the estimated slip velocity, our friction measurements are therefore all conducted in the low speed regime.

Supplementary Fig. 7 shows a comparison of two repeated frictional force measurements at two different scanning speeds. It is seen that the two force curves overlap with each other in most positions, indicating that the measured $F(x)$ and the resulting stick-slip events are not sensitive to the change of scanning speeds used in the experiment. There are a few locations where the two force curves show some deviations. These deviations are mostly associated with small-sized slips and can be attributed to the stochastic slip dynamics. As will be shown in Sec. II.F below, the obtained statistical properties of stick-slip friction at meso-scale are robust and do not change with the scanning speeds in the low speed regime.

II. SUPPLEMENTARY DISCUSSION ON DATA ANALYSIS

A. Identification of slip events

Supplementary Fig. 8 shows a typical frictional force loop obtained when the 2D probe is pulled back and forth for a cycle. Before the scan starts, the 2D probe is in static contact with the sandpaper surface under a normal load $N = 600$ nN. As the probe is pulled to advance ($\rightarrow$) (or recede ($\leftarrow$)), the resulting static frictional force F increases (or decreases) quickly and linearly with the sandpaper displacement x and gives rise to a straight line on the left (or right) side of the force loop. When the pulling force reaches a critical value F_c, the solid interface depins and the 2D probe begins its stick-slip motion, as shown by the top (or bottom) horizontal fluctuating force curve.

To identify the beginning and end of each slip event, we take a differential of the measured advancing frictional force $F(x)$ and obtain the force differential curve $dF(x) = F(x_i) - F(x_{i+1})$, where i and $i+1$ are two adjacent sampling points of the measured $F(x)$, as shown in Supplementary Fig. 9(a). Because the sampling rate is 1 kHz, the time step between the two adjacent sampling points, $\Delta t = 1$ ms, is held constant. The corresponding spatial step of the displacement is $\Delta x = x_i - x_{i+1} \simeq U\Delta t$. For $U = 100$ nm/s, we have $\Delta x = 0.1$ nm. Because U is not held strictly constant (having small fluctuations), $\Delta x = 0.1$ nm is true only on the average sense. As shown in Supplementary Fig. 9(b), individual slip events can be identified by the sharp spikes in the measured $dF(x)$ curve. On average, a single slip (spike) lasts for about 5 ms (5 sampling points).

To discriminate the noise in the measured $dF(x)$ curve, we set a threshold value ϵ, so that all the spikes in the measured $dF(x)$ with an amplitude $dF(x) > \epsilon$ are counted as slip events. The threshold value ϵ is deter-

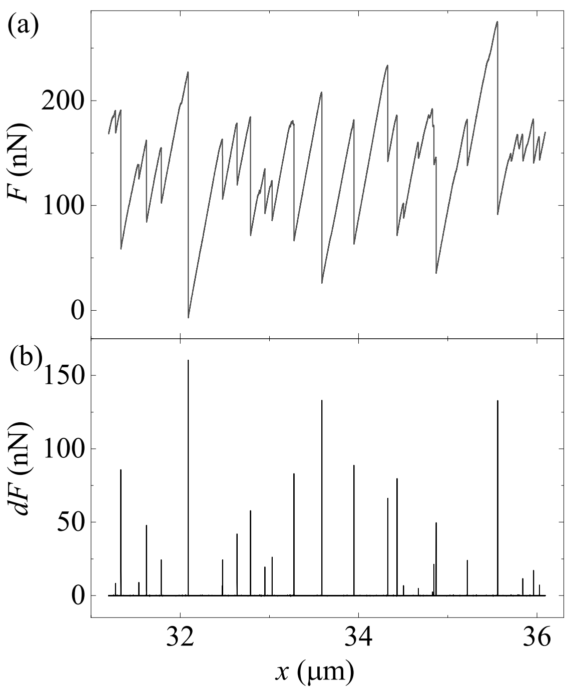

Supplementary Fig. 9. **Identification of slip events from a measured force trajectory.** (a) A zoom-in plot of the measured advancing frictional force $F(x)$ in Supplementary Fig. 8. (b) Corresponding force differential $dF(x)$ between two adjacent sampling points of the measured $F(x)$ in (a).

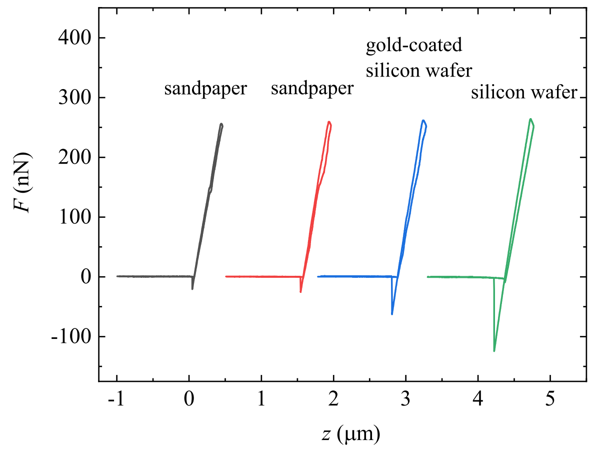

Supplementary Fig. 10. **Measurements of interfacial adhesion.** Plots show the measured vertical (contact) force as a function of vertical traveling distance of the cantilever when the scanning probe approaches and then recedes from different substrates. The black and red curves are obtained, respectively, for the quasi-1D probe and 2D probe over the sandpaper surface. The blue and green curves are obtained for the 2D probe over the gold-coated and bare silicon wafer surfaces, respectively. All the measurements are made at a constant speed of 2 μm/s. For clarity, the red, blue and green curves are shifted to the right by 1.5 μm (red), 2.9 μm (blue) and 4.4 μm (green), respectively.

mined as follows. (i) We first calculate the RMS value of $dF(x)$ and remove those data points whose amplitude $dF(x)$ is larger than the RMS value. (ii) We then repeat this procedure to the remaining data points for three times so that the final RMS value converges to a constant value, which is the intrinsic noise of the system made up mainly by the environmental vibration and electronic noise of the AFM. (iii) The threshold value ϵ is set as twice the final RMS value. The obtained threshold value ϵ is usually in the range of 0.2–0.5 nN, which varies slightly with the cantilever used and the environmental noise. Once the slip events are identified, the force at the beginning of each slip event is recorded as the maximal (depinning) force F_c and the force drop between the beginning and end of each slip event is recorded as the force release δf associated with that slip event.

B. Determination of interfacial adhesion

Supplementary Fig. 10 shows four typical contact-force-versus-distance curves $F(z)$ measured when the scanning probes approach and then recede from different substrates. The measured $F(z)$ all increases linearly with the cantilever displacement z and the slope gives the spring constant of the cantilever in the normal direction. The measured force loops in the advancing and receding directions overlap quite well with little hysteresis. It is seen from the black and red curves that when the scanning probe detaches from the sandpaper surface, there is a pull-off force resulting from the adhesion between the probe and sandpaper. This adhesion force is about the same for the quasi-1D and 2D probes over the sandpaper surface. By repeating the force measurements at 10 different locations on the same substrate, we obtain the mean value and the standard deviation of the adhesion force to be 21± 6 nN (for 1D probe/sandpaper) and 23±7 nN (for 2D probe/sandpaper), respectively. This adhesion force is small when compared with the normal load N used in the experiment, which is in the range of 200–600 nN. On the other hand, the adhesion force between the 2D probe and the smooth gold-coated silicon wafer surface as well as the bare silicon wafer surface increases significantly with the adhesion force equal to 59±4 nN (for 2D probe/gold-coated silicon wafer) and 125±2 nN (for 2D probe/silicon wafer), respectively. We, therefore, conclude that the reduction of adhesion between the scanning probe and sandpaper results mainly from the rough and chemically inert surface of the sandpaper.

C. Determination of the friction coefficient μ_c

Supplementary Fig. 11 shows how the mean value of the maximal lateral force F_c needed to trigger the lo-

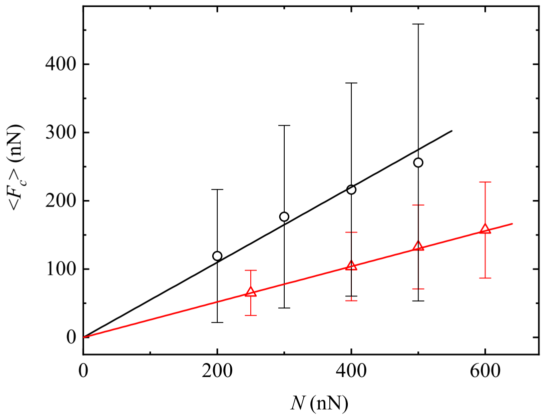

Supplementary Fig. 11. **Measurements of the friction coefficient** μ_c**.** The two sets of data are measured for the quasi-1D probe (black circles) and 2D probe (red triangles) at a scanning speed $U = 100$ nm/s. The error bars show the standard deviation of the measured F_c. The solid lines show the linear fits, $\langle F_c \rangle = \mu_c N$, to the data points with $\mu_c = 0.55 \pm 0.03$ for the quasi-1D probe and $\mu_c = 0.26 \pm 0.02$ for the 2D probe.

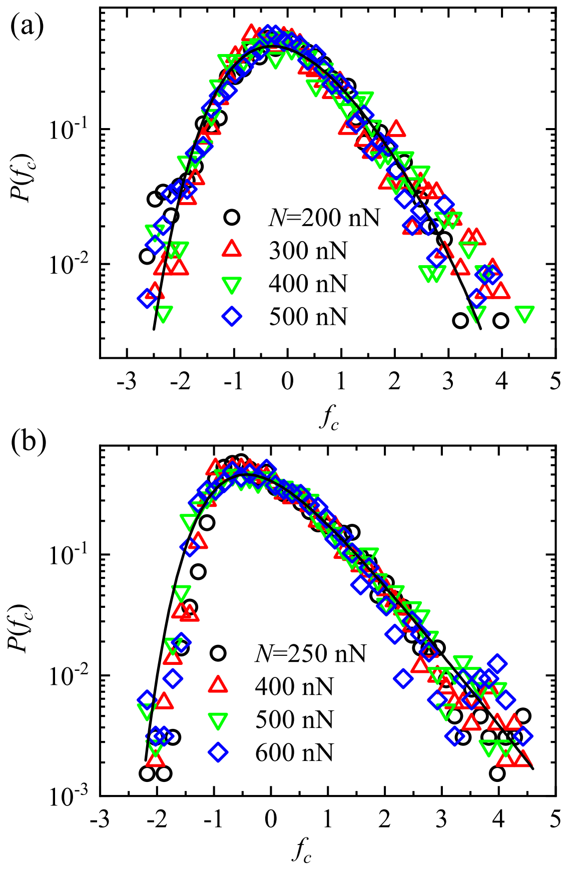

Supplementary Fig. 12. **PDFs of the normalized maximal force under different normal loads.** The measurements are made for the quasi-1D probe (a) and 2D probe (b) at the same scanning speed $U = 100$ nm/s. The black solid lines show the fits of the GEV distribution (Eq. (5)) to all the data points with (a) $\xi = -0.13 \pm 0.06$ and (b) $\xi = -0.03 \pm 0.05$.

cal slips changes with the normal load N. The relatively large error bars result from the large fluctuations of the maximal frictional force measured in the stick-slip regime. It is seen that the obtained $\langle F_c \rangle$ is linearly proportional to N, indicating that the Amontons' law of friction [11] still holds for stick-slip friction. The solid lines in Supplementary Fig. 11 show the linear fits, $\langle F_c \rangle = \mu_c N$, to the data points with $\mu_c = 0.55 \pm 0.03$ for the quasi-1D probe and $\mu_c = 0.26 \pm 0.02$ for the 2D probe.

D. Normal load effects on the frictional force statistics

1. *PDFs of the normalized maximal force* f_c

The generalized extreme value (GEV) distribution is given in Eq. (1) of the main text and is repeated here for ease of reference [12]:

$$P(f_c) = \frac{1}{\beta}\left(1 + \xi \frac{f_c - \mu}{\beta}\right)^{-(1+1/\xi)} \exp\left[-\left(1 + \xi \frac{f_c - \mu}{\beta}\right)^{-1/\xi}\right], \tag{5}$$

where f_c is a random variable. In the general case in which f_c is not normalized, Eq. (5) has three independent parameters: a location parameter μ, a scale parameter β and a shape parameter ξ. The location parameter μ and scale parameter β are approximately the mean and standard deviation of f_c. The shape parameter ξ determines the tail shape of the GEV distribution, with $\xi = 0$ for an exponential tail, $\xi > 0$ for a heavy tail and $\xi < 0$ for a short or no tail. When f_c is normalized with a zero mean and unity variance, as stated in Eq. (1) of the main text, the three parameters in Eq. (5) are bounded by the two normalization constraints, $\langle f_c \rangle = \mu + \beta[\Gamma(1-\xi) - 1]/\xi = 0$ and $\sigma^2_{f_c} = \beta^2[\Gamma(1-2\xi) - \Gamma^2(1-\xi)]/\xi^2 = 1$, where $\Gamma(\cdots)$ is the Gamma function. In this case, Eq. (5) has only one independent parameter. By choosing ξ as the independent parameter, we have $\beta = |\xi|/\sqrt{\Gamma(1-2\xi) - \Gamma^2(1-\xi)}$ and $\mu = \beta[1 - \Gamma(1-\xi)]/\xi$.

When $\xi = 0$, Eq. (5) can be simplified by taking the limit $\xi \to 0$, i.e.,

$$\lim_{\xi \to 0} P(f_c) = \frac{1}{\beta_0} \exp\left(-\frac{f_c - \mu_0}{\beta_0} - e^{-(f_c - \mu_0)/\beta_0}\right), \tag{6}$$

where $\beta_0 = \sqrt{6}/\pi$ and $\mu_0 = -\sqrt{6}\gamma/\pi$ with $\gamma \simeq 0.577$ being the Euler's constant. Equation (6) represents a special case of the GEV distribution (with $\xi = 0$) and is called the Gumbel distribution, which has a long exponential tail.

Supplementary Figs. 12(a) and 12(b) show the measured PDFs $P(f_c)$ of the normalized maximal force f_c

for the quasi-1D probe and 2D probe, respectively, under different normal loads N. Here we define $f_c = (F_c - \langle F_c \rangle)/\sigma_{F_c}$ with $\langle F_c \rangle$ and σ_{F_c} being, respectively, the mean and standard deviation of F_c. It is seen that for the same probe, the measured PDFs $P(f_c)$ at different N all collapse onto a master curve, which can be well described by the GEV distribution in Eq. (5). The black solid lines in Supplementary Fig. 12 show the fits of Eq. (5) to the data points, from which we find $\xi = -0.13 \pm 0.06$ for the quasi-1D probe and $\xi = -0.03 \pm 0.05$ for the 2D probe. The error bar of the fitting parameters quoted here represents the confidence interval that we obtain from the fitting. The obtained values of ξ for the two probes are both close to zero, suggesting that the obtained PDFs $P(f_c)$ are close to the Gumbel distribution in Eq. (6). Supplementary Fig. 12 thus suggests that the GEV (or Gumbel) distribution is a universal description of the depinning force for local slips, which is invariant with the normal load N in the stick-slip regime studied.

2. *PDFs of the slip length δx_s*

Supplementary Figs. 13(a) and 13(b) show the measured PDFs $P(\delta x_s)$ of the slip length δx_s for the quasi-1D probe and 2D probe, respectively, under different normal loads N. The slip length δx_s is the force release δf normalized by the static spring constant k_0, i.e., $\delta x_s = \delta f / k_0$. It is seen from Supplementary Fig. 13(a) that the measured PDFs $P(\delta x_s)$ for the quasi-1D probe at different values of N all collapse onto a master curve, which can be well described by the power-law distribution

$$P(\delta x_s) \sim (\delta x_s)^{-\tau}, \tag{7}$$

for all values of δx_s smaller than a cutoff slip length $\delta x_s^* \simeq 800$ nm. The fitted value of the power-law exponent is $\tau = 1.12 \pm 0.10$ (black solid line) and the error bar quoted here represents the confidence interval that we obtain from the fitting. Supplementary Fig. 13(a) thus suggests that the observed power-law distribution of the slip length δx_s for the quasi-1D probe is invariant with the normal load N in the stick-slip regime studied.

It is seen from Supplementary Fig. 13(b) that the measured PDFs $P(\delta x_s)$ for the 2D probe reveal some weak dependence on N for the two sets of data with $N \leq 400$ nN. These two sets of data can still be fitted to Eq. (7) but the fitted value of τ decreases slightly with increasing N from $\tau = 0.92$ at $N = 250$ nN to $\tau = 0.82$ at $N = 400$ nN. Furthermore, the cutoff slip length δx_s^* increases gradually with N from $\delta x_s^* \simeq 160$ nm at $N = 250$ nN to $\delta x_s^* \simeq 270$ nm at $N = 400$ nN. For $N \geq 500$ nN, the measured PDFs $P(\delta x_s)$ reach to an asymptotic form and do not change with N any more. The two sets of data can be well described by a common power-law distribution, as shown in Eq. (7), with $\tau = 0.72 \pm 0.10$ for all values of δx_s smaller than the cutoff slip length $\delta x_s^* \simeq 400$ nm. These results suggest that the observed power-law distribution of the slip length δx_s for the 2D probe becomes invariant with the normal load N, when N is large enough so that the 2D probe is in an "optimal" contact with the sandpaper and can feel its entire roughness landscape.

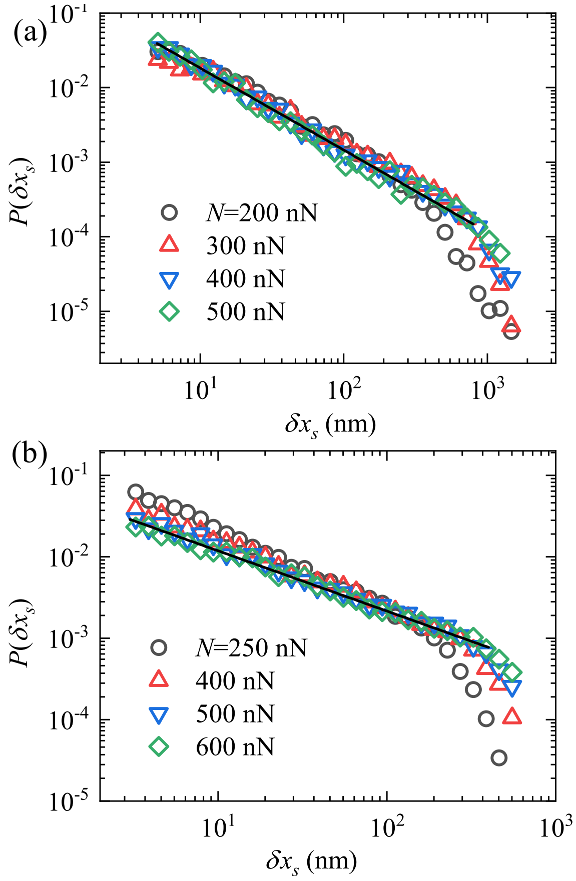

Supplementary Fig. 13. **PDFs of the slip length under different normal loads.** The measurements are made for the quasi-1D probe (a) and 2D probe (b) at the same scanning speed $U = 100$ nm/s. The black solid lines show the power-law fits, $P(\delta x_s) \sim (\delta x_s)^{-\tau}$, to the data points with $\tau = 1.12 \pm 0.10$ (a) and $\tau = 0.72 \pm 0.10$ (b). The power-law fit in (a) is made to all the four sets of data points. The power-law fit in (b) is made only to the two sets of data points with $N = 500$ nN and $N = 600$ nN.

3. *PDFs of the local force gradient k'*

Supplementary Figs. 14(a) and 14(b) show the measured PDFs $P(k')$ of the local force gradient $k' \equiv dF_i(x_s)/dx_s$ for the quasi-1D probe and 2D probe, respectively, under different normal loads N. Here $F_i(x_s)$ is the random pinnning force field sampled by the solid interface and k' is extracted from the measured dynamic spring constant k using the equation $k' = kk_0/(k_0 - k)$ (see Eq. (13) below). In the plot, k' is normalized by the static spring constant k_0, which is independent of N.

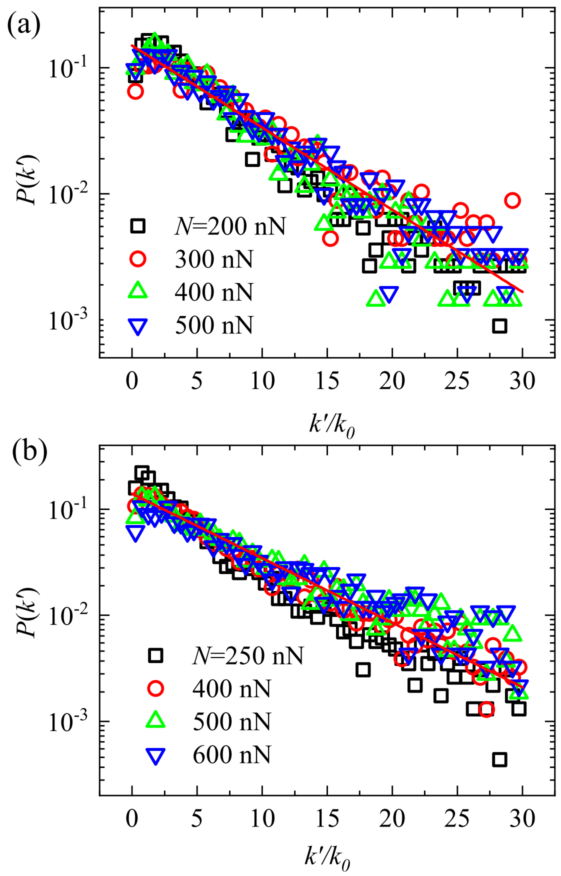

Supplementary Fig. 14. **PDFs of the local force gradient under different normal loads.** The value of k' is extracted from the measured dynamic spring constant k using Eq. (13) and is normalized by the static spring constant k_0. The measurements are made for the quasi-1D probe (a) and 2D probe (b) at the same scanning speed $U = 100$ nm/s. The red solid lines show the fits of Eq. (8) to the data points with (a) $b = 0.14 \pm 0.02$ and (b) $b = 0.14 \pm 0.02$.

It is seen that for the same probe, the measured PDFs $P(k')$ at different values of N all collapse onto a master curve within the statistical error and can be described by the exponential distribution

$$P(k'/k_0) = be^{-b(k'/k_0)}, \tag{8}$$

where the decay rate b is a fitting parameter. The red solid lines show the fits of Eq. (8) to the data points, from which we find $b = 0.14 \pm 0.02$ (or $\langle k' \rangle / k_0 = 7.1$) for both the quasi-1D probe and the 2D probe.

E. PDFs of f_c, δx_s and k at the high-load limit

To illustrate the different behaviors of stick-slip friction between the intermediate and high-load regimes, we consider the evolution of asperity configurations at the contact interface with increasing normal load N. Supplementary Fig. 15 shows a sketch of the contact geometry between a deformable flat surface (e.g., the scanning probe with a Young's modulus of ~ 3 MPa) and a rigid rough surface (e.g., the sandpaper with a Young's modulus of ~ 170 GPa). In the low-load regime, the asperities (or microcontacts) at the interface are dilute and their number increases with the normal load N [13]. When the asperities become dense enough, further increase of N introduces interactions and coalesce between nearby asperities. As a result, the number of asperities at the interface starts to decrease with N and consequently the incident rate of slips is reduced, as shown in Figs. 1(e) and 1(f) of the main text. In fact, the effect illustrated in Supplementary Fig. 15 should apply to any normal load, so long as the rough surfaces has a broad range of asperity sizes and separations (see Sec. I.A). Under small normal loads, only those asperities with a very close separation can interact with each other. As the normal load increases, asperities with a relatively large separation can get in touch with each other due to their elastic and (or) plastic deformations. In other words, the coalescence effect is enhanced with an increasing N.

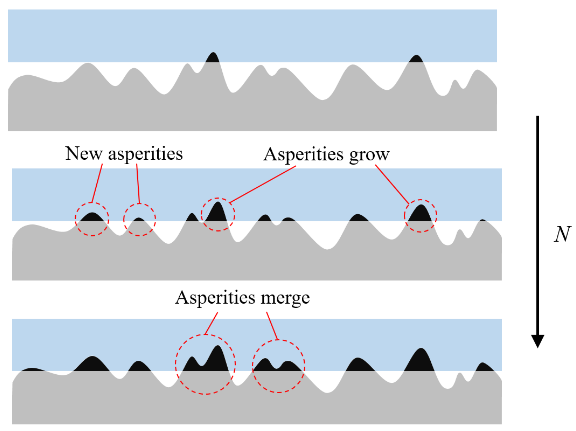

Supplementary Fig. 15. **Evolution of contact geometry with increasing normal load N.** The blue shaded area represents a deformable flat surface and the grey shaded area represents a rigid rough surface.

Assuming the actual contact area A_r is $\sim 1\%$ of the apparent contact area $A_a = 12 \times 12$ μm$^2 = 1.44 \times 10^{-10}$ m^2, we find the effective stress σ_N exerted on the microcontacts at the normal load $N = 2400$ nN to be, $\sigma_N = N/A_r \simeq 1.7$ MPa. This value of σ_N is close to the yield stress of the UV cured glue (estimated as the Young's modulus for a 100% strain). Supplementary Fig. 15 thus provides an argument, which extends the Greenwood and Williamson model [13] for interacting asperities in the high-load regime. The evolution of the contact geometry with an increasing N also implies a continuous change of the random pinning force field. Microscopically, because the local pinning sites in the high-load regime become stronger, multi-asperity slips where the entire scanning probe crosses over a number of asperities in a single slip event are more likely to occur, which also reduces the in-

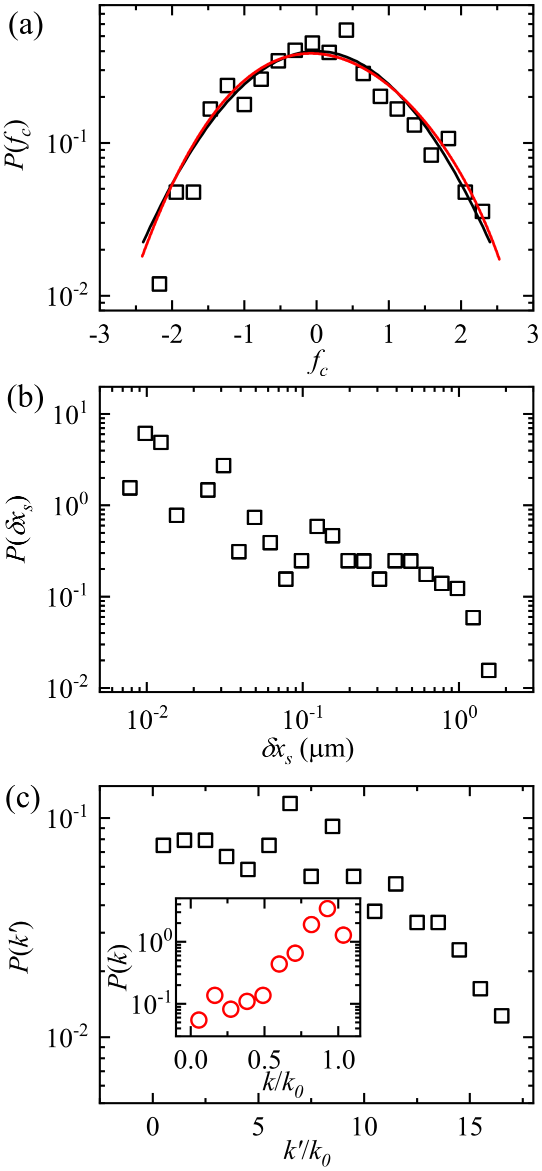

Supplementary Fig. 16. **Statistical properties of the measured stick-slip friction at the high load limit.** (a) Measured PDF $P(f_c)$ of the normalized maximal force f_c. The red solid line shows a fit of the GEV distribution (Eq. (5)) to the data points with $\xi \simeq -0.26$. The data can also be described by a (Gaussian) normal distribution (black solid line). (b) Measured PDF $P(\delta x_s)$ of the slip length δx_s. (c) Measured PDF $P(k')$ of the local force gradient k'. The value of k' is extracted from the measured dynamic spring constant k using Eq. (13) and is normalized by the static spring constant k_0. Inset shows the measured PDF $P(k)$ of the normalized dynamic spring constant k/k_0. All the measurements are made using the 2D probe at the normal load $N = 2400$ nN and scanning speed $U = 100$ nm/s.

cident rate of slips. A similar effect of multi-asperity slips has been observed in nanotribology [14]. As a result, both the effects of asperity coalescence and multi-asperity slips are possible reasons for the observed stick-slip behavior in the high-load regime.

The vertical deflection Δz of the cantilever at the normal load $N = 2400$ nN can be estimated as, $\Delta z = N/k_n \simeq 3.2$ μm, where $k_n = 0.74$ N/m is the spring constant of the cantilever in the normal direction. The cantilever used in the experiment has a rectangular shape and its length $L = 300$ μm. The resulting strain of the cantilever is $\Delta z/L \simeq 1.1\%$. For this small value of strain, vertical deformations of the cantilever are in the linear response regime and their nonlinear effect is negligible. The measurement of the frictional force, on the other hand, uses the lateral deflection Δx of the cantilever, which is independent of Δz. It is seen from Fig. 1(f) in the main text that the measured frictional force $F(x)$ in the stick state changes linearly with the traveling distance x, indicating that the lateral deformations of the scanning probe are also in the linear response regime. Slight deviations of the measured $F(x)$ from its linear dependence on x are observed when the measured $F(x)$ reaches its maximal value before slips take place. We believe that this is caused by the partial slip (or plastic deformation) of the local contacts but not by the nonlinear effect of the hanging-beam probe. This is because a similar behavior is also observed when the value of N is in the intermediate range (200–600 nN), in which the amplitude of the measured $F(x)$ is reduced by more than 4 times, and also in other experiments as reported in Ref. [15].

Supplementary Figs. 16(a), 16(b) and 16(c) show, respectively, the PDFs of the three quantities, f_c, δx_s and k', measured at the high load limit, $N = 2400$ nN. The corresponding force trajectories are shown by the red curve in Fig. 1(e) of the main text. The measured $P(f_c)$ in Supplementary Fig. 16(a) has a nearly symmetric shape, which can be described equally well by either the GEV distribution in Eq. 5 with a large value of $\xi \simeq -0.26$ (red solid line) or a (Gaussian) normal distribution (black solid line). The measured $P(f_c)$ at the high load limit is very different from that obtained at the intermediate values of N, which follows the GEV distribution with a small value of ξ and a long tail for the positive values of f_c. Note that the obtained values of f_c in Supplementary Fig. 16(a) are distributed in a narrower range [-2, +2] compared with that measured at the intermediate values of N, suggesting that the variation range of the resulting f_c at the high load limit is decreased.

The measured $P(\delta x_s)$ in Supplementary Fig. 16(b) does not show a prominent power-law distribution, as that obtained at the intermediate values of N. It reveals a noisy decay for small values of δx_s (< 0.1 μm), followed by a plateau region when δx_s is in the range between 0.1 μm and 1 μm. This behavior of $P(\delta x_s)$ suggests that at the high load limit, slips tend to have larger sizes compared with those obtained at the intermediate values of N.

The measured $P(k')$ in Supplementary Fig. 16(c) does not follow the exponential distribution, as that obtained at the intermediate values of N. It reveals a plateau region for small values of k'/k_0 (< 8), followed by a rapid

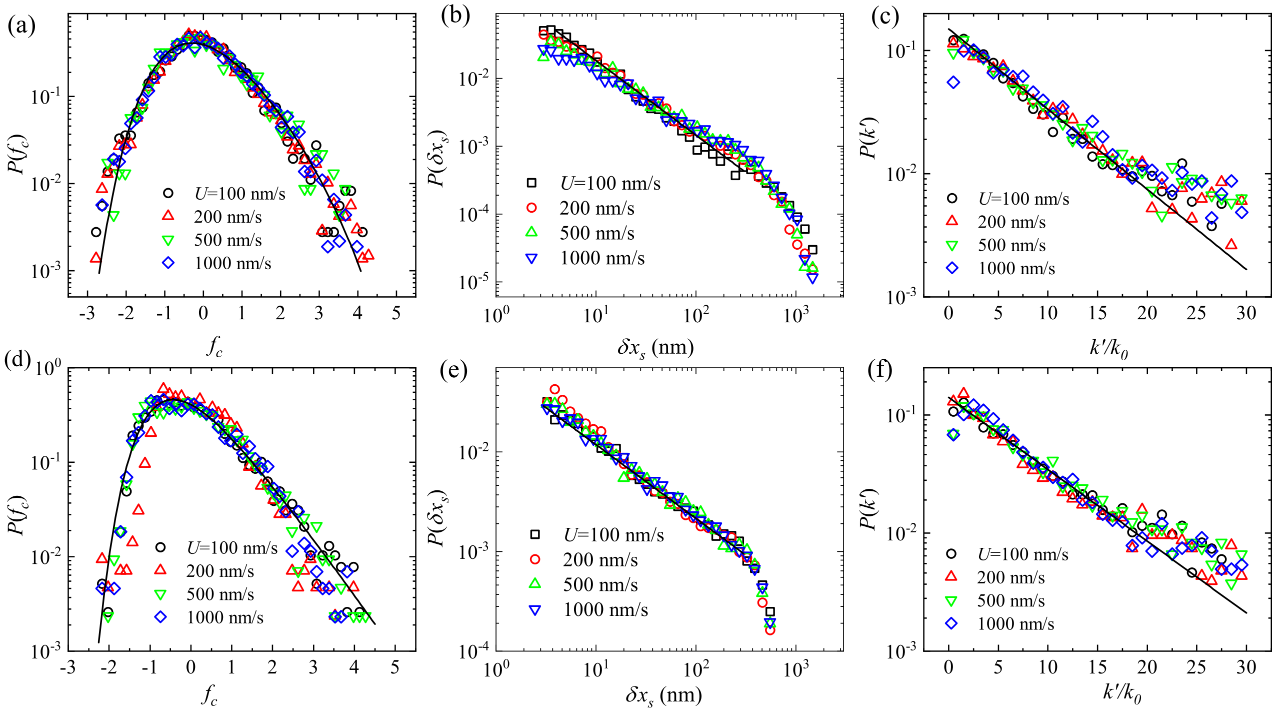

Supplementary Fig. 17. Statistical properties of the measured stick-slip friction at different scanning speeds. The top panels (a,b,c) are measured using the quasi-1D probe at a normal load $N = 500$ nN and the bottom panels (d,e,f) are obtained using the 2D probe at a normal load $N = 500$ nN. The solid lines in (a) and (d) are the fits of the GEV distribution in Eq. (5) to the data with (a) $\xi = -0.13 \pm 0.06$ and (d) $\xi = -0.03 \pm 0.05$. The solid lines in (b) and (e) are the fits of the power-law distribution in Eq. (7) to the data with (b) $\tau = 1.12 \pm 0.10$ and (e) $\tau = 0.72 \pm 0.10$. The solid lines in (c) and (f) are the fits of the exponential distribution in Eq. (8) to the data with (c) $b = 0.14 \pm 0.02$ and (f) $b = 0.14 \pm 0.02$.

decay for larger values of k'/k_0 (> 8). This behavior of $P(k')$ suggests that the scanning probe experiences a stronger pinning field at the high load limit and thus produces a heavier distribution than the exponential one. This is consistent with the observation that the measured PDF $P(k)$ (inset of Supplementary Fig. 16(c)) of the dynamic spring constant k has a higher weight at large k ($\geq 0.6k_0$) compared with that measured at the intermediate values of N.

The above results indicate that the stick-slip friction at large values of N does not follow the three statistical laws as observed at the intermediate values of N. This is caused by a large reduction both in numbers and in strengths of the asperities at the contact when it is under a large normal load. This finding further suggests that the stick-slip friction under the intermediate normal load is at a "critical state", in which the contact between the two solid surfaces has an adequate number of random asperities and each individual asperity can locally generate a unique pinning to the moving interface. Under this condition, the scanning probe explores the entire landscape of the pinning force field at the contact with the rough sandpaper surface, so that the random stick-slip friction is characterized by the three statistical laws as reported in the main text.

F. Scanning speed effects on the frictional force statistics

Supplementary Fig. 17 shows the measured PDFs of the normalized maximal force f_c (a,d), slip length δx_s (b,e) and local force gradient k' (c,f) for different scanning speeds U. It is seen that the measured PDFs at different values of U all collapse onto a single curve, suggesting that the functional form of the measured $P(f_c)$, $P(\delta x_s)$ and $P(k')$ is invariant with the scanning speed U studied. This conclusion applies to all the friction measurements conducted by using both the quasi-1D and 2D probes. The measured $P(f_c)$, $P(\delta x_s)$ and $P(k')$ are well described, respectively, by the GEV distribution in Eq. (5), the power-law distribution in Eq. (7) and the exponential distribution in Eq. (8) with the same fitting parameters as those reported in the main text. The results shown in Supplementary Fig. 17 thus demonstrate that the observed statistical laws of stick-slip friction at meso-scale are robust and do not change with the scanning speed U in the low speed regime.

G. Frictional force fluctuations at mesoscale

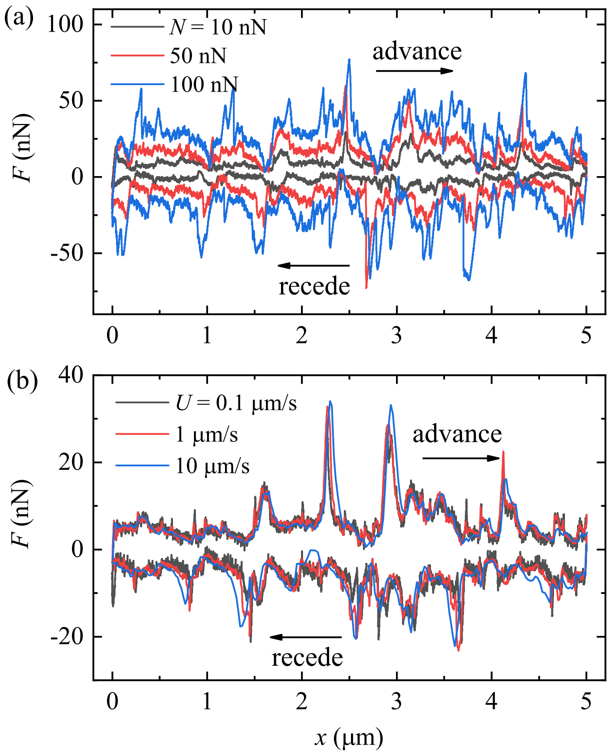

Supplementary Fig. 18. **Measured frictional force loops under very small normal loads and at the low speed limit.** The measurements are made when the 2D scanning probe moves forward (advance) and backward (recede) on the same sandpaper surface for a cycle under two types of conditions: (a) constant scanning speed $U = 100$ nm/s but different normal loads N, (b) same normal load $N = 20$ nN but different scanning speeds U.

in the small load regime ($N < N_m$)

To further understand the low-load behavior of the frictional force at mesoscale, we conduct additional measurements of the frictional force in the small load regime ($N < N_m$ with $N_m = 124$ nN, see the main text). Supplementary Fig. 18(a) shows the frictional force loops under three different normal loads in the small load regime. At $N = 100$ nN, the force trajectory shows continuous fluctuations with a few abrupt changes (slips) occasionally. This kind of force trajectories are considered as smooth sliding, because the scanning probe only feels a weak pinning field with a finite number of predominantly small asperities, which are too small to cause stick-slip instabilities. As N decreases, the number of small asperities decreases (see Supplementary Fig. 15) and so does the magnitude of the measured force fluctuations. The residual rare slip events become fewer and eventually disappear at $N = 10$ nN. Note that the hysteresis between the mean frictional forces in the advance direction and in the receding direction also decreases with decreasing N. Such a reduction of friction-induced hysteresis with decreasing normal load was also observed in atomic friction at the single contact limit [16].

Supplementary Fig. 18(b) shows how the measured frictional force loops change with the scanning speed U when the normal load is held at a small constant value of $N = 20$ nN. It is seen that the measured frictional force loops do not reveal any significant speed-dependence in the small load regime. This result is in agreement with a general trend that was observed for rough surfaces [15, 17] that the dynamic friction coefficient in steady sliding is roughly a constant or decreases slightly with increasing U when U is small, with a nonzero quasi-static limit as $U \to 0$. This general trend is also confirmed in the high-load regime, as shown in Supplementary Fig. 17. The result shown in Supplementary Fig. 18(b), however, does not agree with those obtained in atomic friction at the single contact limit, in which the frictional force goes to zero as $U \to 0$ [16, 18].

For weak pinning fields, such as those in atomic friction with a characteristic energy barrier E_b and lattice constant λ, the stick-slip instability gives rise to a nonzero frictional force of the order of E_b/λ at the low sliding speed limit [18, 19]. For even weaker pinning fields (or smaller normal load N) where the stick-slip instability does not occur, the sliding tip can follow the corrugated surface landscape and the effect of the energy barrier E_b may be averaged out with a near-zero frictional force at the limit of low sliding speed and single elastic contact [16, 18]. The situation is changed when the apparent contact area contains a finite number of micro-contacts or asperities, which are randomly distributed both in space and in size. In this case, the effect of the energy barriers associated with different asperities cannot be averaged out coherently, and thus gives rise to a non-zero frictional force at the quasi-static limit as $U \to 0$.

As indicated in Eq. (14) below, the lateral force measured by AFM is a sum of the viscous drag force and the pinning (frictional) force. The former is a linear function of U, whereas the latter usually does not change much with U, as discussed above. In most dry friction experiments, the viscous drag force at the low speed limit is so small compared with the frictional force and thus is neglected [20, 21]. The results shown in Supplementary Fig. 18(b) thus suggest that force fluctuations in the small load (or steady sliding) regime result primarily from the low-load friction at the mesoscale contact, which contains a finite number of asperities and gives rise to a weak pinning force that has a non-zero value even at the low speed limit.

III. SUPPLEMENTARY DISCUSSION ON THEORETICAL MODEL

A. Frictional force and effective spring constant in the stick state

The total frictional force at a solid interface can be expressed in terms of the asperity-induced heterogeneous

shear stress $\sigma(x, y; x_s)$ at the interface

$$F_i(x_s) = \iint_{A(x_s)} \sigma(x, y; x_s) dx dy = \int_{L_y} h(y; x_s) dy, \quad (9)$$

where x, y are the Cartesian coordinates of the contact interface of area $A(x_s)$ and x_s is the coordinate of the center-of-mass position of the interface along the moving direction x. The shear stress $\sigma(x, y; x_s)$ varies with the position of the scanning probe on the sandpaper because of the surface roughness and thus it depends on x_s. By introducing an effective interfacial tension $h(y; x_s) = \int_{L_x} \sigma(x, y; x_s) dx$, the motion of the scanning probe can be envisioned as a "contact line" sweeping over the substrate (sandpaper) along the x direction, as indicated by the second equality of Eq. (9). In the above, L_x and L_y are, respectively, the boundary length of the contact area in the x and y direction and thus we have $A(x_s) = L_x L_y$. Equation (9) indicates that $F_i(x_s)$ can be thought as an asperity-induced random pinning force field so that the "moving contact line" will feel varying pinning strengths along x_s.

With Eq. (9), we write the force release δf during a slip as

$$\delta f = \int_0^{L_y} [h(y; x_s) - h(y; x_s + \delta x_s)] dy, \quad (10)$$

where δx_s is the slip distance. By taking Taylor expansion of the integrand in Eq. (10) to the first order with the resulting $\langle \partial h/\partial x_s \rangle_{\delta A}$ being averaged over the area δA enclosed between x_s and $x_s + \delta x_s$, Eq. (10) can be rewritten as

$$\delta f \simeq - \left\langle \frac{\partial h}{\partial x_s} \right\rangle_{\delta A} \iint_{\delta A} dx_s dy = k \delta x_s \simeq k_0 \delta x_s, \quad (11)$$

where $k = -\langle \partial h/\partial x_s \rangle_{\delta A} L_y$ is an effective spring constant and $\delta x_s = (1/L_y) \iint_{\delta A} dx_s dy$. At the onset of a local slip, the local (downward) slope k of the pinning force field $F_i(x_s)$ becomes equal to the spring constant k_0 of the pulling spring (namely, the static spring constant k_0 of the scanning probe), so that the pulling force will exceed the pinning force as the scanning probe moves forward slightly. As a result, the scanning probe will accelerate and a local slip takes place. The last relation in Eq. (11) thus establishes a direct connection between the measured force release δf and the slip length δx_s.

Supplementary Fig. 19 illustrates the onset condition $k = k_0$ graphically. The force variations from A to E represent a complete stick-slip cycle. At Point A, the elastic pulling force

$$F_e(x_0; x_s) = k_0(x_0 - x_s), \quad (12)$$

at the pulling point $x_0^{(1)}$ (black solid line) is balanced by the frictional (or pinning) force field $F_i(x)$ (red solid curve) so that the red and black solid lines have a common intersection at Point A and the center-of-mass (CoM) of the scanning probe is in the stick phase at x_A. As the pulling point $x_0^{(1)} = Ut$ moves continuously to the right at a constant speed U, the intersection point moves from A to B following the red curve and correspondingly the CoM moves from x_A to x_B. Along the moving path $A \to B$, the system is said to be in the stick state, because the displacement of the CoM ($= x_B - x_A$) is much smaller than that of the pulling point ($= x_0^{(2)} - x_0^{(1)}$). When the CoM moves to x_B, where the local (downward) slope dF_i/dx_s of the force field becomes equal to $-k_0$, the pulling force $F_e(x_0; x_s)$ can no longer be balanced by the pinning force $F_i(x_s)$ and the system becomes unstable and slip occurs.

The end position of the CoM after the slip depends on the system damping. For an over-damped system, the CoM will jump to x_C and stay there. For a zero-damping system, the CoM will jump to x_D and then oscillates between x_D and x_C, such that the grey shaded area under the black dashed line is equal to the blue shaded area above the black dashed line (by ignoring the dissipation generated by the mean frictional force). For a system with a moderate damping, the CoM will jump to a posi-

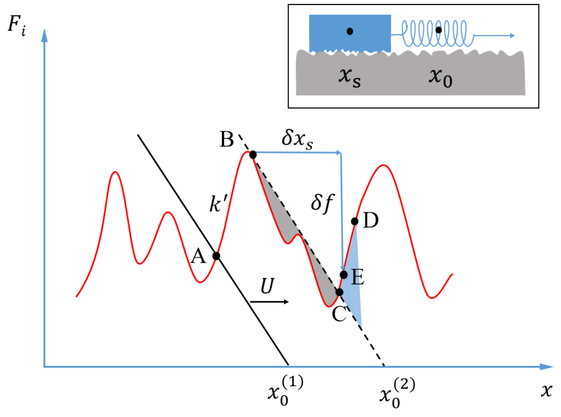

Supplementary Fig. 19. **A diagram showing the stick-slip motion in a random pinning force field.** The red curve represents the 1D random pinning force field $F_i(x)$. The black solid line with a slope $-k_0$ represents the elastic pulling force $F_e(x_0; x_s)$, as shown in Eq. (12), with x_0 being the position of the pulling point and x_s being the position of the center-of-mass (CoM, black dots) of the scanning probe, as determined by the intersection of the red and black solid lines. As the pulling point moves at a constant speed U from $x_0^{(1)}$ to $x_0^{(2)}$, the black solid line moves to the right (black dashed line) and correspondingly the CoM moves from x_A to x_B. At the onset of slip, the CoM is at x_B, where the local slope of the pinning force field dF_i/dx_s becomes equal to $-k_0$. The end position of the CoM after the slip depends on the system damping, as explained in the text. Inset (top right) shows a sketch of the spring-block model for the CoM moving over a rough surface. Note that the pulling point x_0 is not at the free end of the spring but is located somewhere in the middle of the spring so that the elastic pulling force takes the form given in Eq. (12).

tion between x_C and x_D, say x_E, and then undergoes a damped oscillation and gradually drifts toward x_C, until the remaining kinetic energy is fully dissipated. Because the slip occurs rapidly, the pulling point remains at $x_0^{(2)}$ during the slip and the displacement of the CoM (or slip length) is thus given by $\delta x_s = x_E - x_B$. Because of friction at the solid interface, the actual stick-slip motion is always influenced by some level of damping. As will be shown below, the use of the relation, $\delta x_s = \delta f/k_0$, to estimate the slip length, which is valid for over-damped systems, is indeed a good approximation for our system with an accuracy better than 90%.

By taking a differential of the stick-state condition, $F_e(x_0; x_s) = F_i(x_s)$, with respect to x_s, we have $dx_s = dx_0/(1+k'/k_0)$, where $k' = dF_i(x_s)/dx_s$ is the local (upward) force gradient (or spring constant) of the pining force field $F_i(x_s)$, as indicated in Supplementary Fig. 19. This relation suggests that the CoM is pinned only partially in the stick state and its movement dx_s is a factor of $1/(1 + k'/k_0)$ smaller than the displacement δx_0 of the pulling point. Furthermore, the instantaneous velocity dx_s/dt of the CoM is smaller than $U = dx_0/dt$ when the CoM moves upwards ($k' > 0$) along $F_i(x_s)$ and is larger than U when it moves downward ($k' < 0$). This incoherent motion between the CoM and the pulling spring gives rise to a fluctuating dynamic spring constant k defined as $k \equiv dF_i(x_s)/dx_0$. By taking a derivative of $F_e(x_0; x_s) = F_i(x_s)$ with respect to x_0, we have

$$k = \frac{k' k_0}{k' + k_0}. \tag{13}$$

Equation (13) indicates that the measured k is an effective spring constant of two springs connected in series, wherein one has a spring constant k_0 and the other has k'.

The onset condition for slip (i.e., $k = k_0$) can be experimentally verified by examining the steady-state condition that the total displacement Δx_s of the CoM (of the scanning probe) over a long run is equal to the total displacement Δx_0 of the pulling point. The latter is just the scanning distance of the AFM scanner, i.e., $\Delta x_0 = 500$ μm, which can be directly read out from the AFM controller. The former contains two parts, i.e., $\Delta x_s = \Delta x_s^{(a)} + \Delta x_s^{(b)}$, where $\Delta x_s^{(a)}$ is the total displacement during the slip and $\Delta x_s^{(b)}$ is the total displacement during the partial slip in the stick state. With these definitions, we have $\Delta x_s^{(a)} = \sum_i (\delta x_s)_i \simeq \sum_i (\delta f_i/k_0)$, where $(\delta x_s)_i$ and δf_i are, respectively, the slip displacement and force drop associated with the ith slip event, and $\Delta x_s^{(b)} = \sum_i (\delta x_0)_i/(1 + k'_i/k_0) \simeq \Delta x_0/(1 + \langle k'/k_0 \rangle)$, where $(\delta x_0)_i$ and k'_i are, respectively, the displacement of the pulling point and the local force gradient of the pinning force during the ith stick event. From the force trajectories obtained at $U = 100$ nm/s and under different normal loads N, we calculate the values of $\Delta x_s^{(a)}$ and $\Delta x_s^{(b)}$, and the final results are summarized in Supplementary Table 1. It is seen that the total displacements Δx_s of the CoM obtained under different experimental conditions are all very close to the scanning distance Δx_0 (with over 91% agreement). This result thus confirms that the relation $\delta x_s \simeq \delta f/k_0$, which is used to calculate Δx_s, indeed gives a good estimate of the slip length from the measured force drop. The small difference between Δx_s and Δx_0 ($< 9\%$) may be attributed to a small underestimation made by using the relation $\delta x_s \simeq \delta f/k_0$, as discussed above, and/or the nonlinear effect of very large slip events.

B. An under-damped spring-block model for the slip dynamics

We now propose an under-damped spring-block model, as illustrated in Supplementary Fig. 19, to explain the observed power-law distribution of the slip length δx_s. The equation of motion of the CoM position $x(t)$ can be written as (here we use x instead of x_s to simplify the notation)

$$m\frac{d^2x}{dt^2} + \gamma\frac{dx}{dt} = k_0(Ut - x) - F_i(x). \tag{14}$$

The first term on the left hand side of Eq. (14) is the inertial term with m being the effective mass of the scanning probe. The second term is a damping term with γ being the damping coefficient. This term is used to describe rapid dissipations in solid friction. This term was first adopted in the original Prandtl and Tomlinson (PT) model [16, 22] and later in many of the follow-up models [15, 23, 24]. On the right hand side of Eq. (14), the first term is the elastic pulling force with k_0 being the static spring constant. The pulling force is exerted by the cantilever and hanging beam moving at a constant speed U. The second term $F_i(x)$ (> 0) is the asperity-induced random pinning (or frictional) force field acting on the CoM. Note that $F_i(x)$ is intrinsically dissipative and its mean value $F_0 = \langle F_i(x) \rangle > 0$ is responsible for keeping the system at a steady state. The constant frictional force F_0 only gives rise to an additional steady-state stretching F_0/k_0 to the pulling spring without changing the fluctuation dynamics. The fluctuating force $F_i(x) - F_0$ is responsible for the dynamics of the scanning probe. It is assumed that $F_i(x)$ (or its fluctuation part) is Brownian correlated, i.e., $\langle |F_i(x) - F_i(x')|^2 \rangle = 2D|x - x'|$, where D is a measure of the fluctuation amplitude of $F_i(x)$. The Brownian correlated random force field has been used previously in the Alessandro-Beatrice-Bertotti-Montorsi (ABBM) model for the over-damped avalanche dynamics [25, 26].

As mentioned in the main text, Eq. (14) is a further extension of the PT model, which has been widely used in the study of dry friction and nanotribology [16, 22]. The PT model was considered as the most successful and influential "minimalistic" model in rationalizing the wealth of nanoscale and mesoscale friction data produced over

Probe	$\Delta x_s^{(b)}$	$N = 300$ nN	$N = 400$ nN	$N = 500$ nN	$N = 600$ nN
		$\Delta x_s^{(a)}$ $(\frac{\Delta x_s}{\Delta x_0})$	$\Delta x_s^{(a)}$ $(\frac{\Delta x_s}{\Delta x_0})$	$\Delta x_s^{(a)}$ $(\frac{\Delta x_s}{\Delta x_0})$	$\Delta x_s^{(a)}$ $(\frac{\Delta x_s}{\Delta x_0})$
quasi-1D	74.6 μm	386.5 μm (92%)	386.4 μm (92%)	379.4 μm (91%)	
2D	61.7 μm		405.4 μm (93%)	430.9 μm (99%)	442.2 μm (101%)

Supplementary Table 1. Obtained values of the total displacement $\Delta x_s^{(a)}$ during the slip under different normal loads N and the total displacement $\Delta x_s^{(b)}$ during the partial slip in the stick state, which is independent of N. The total displacement of the CoM is $\Delta x_s = \Delta x_s^{(a)} + \Delta x_s^{(b)}$, which varies between the quasi-1D probe and 2D probe. The scanning distance of the AFM scanner is $\Delta x_0 = 500$ μm, which is the same for both the quasi-1D and 2D probes.

the last decades [18, 22, 27]. A number of experiments have been conducted and demonstrated the characteristic underdamped features of dry friction. For example, attenuated oscillations after a slip were observed in the friction-force traces obtained using a surface force apparatus with two mica surfaces sliding across a solidified molecular film [28]. Overshoots of a single slip over multiple slip-lengths were found in the lateral force trajectories obtained when a sharp AFM tip slides over a crystalline solid surface [14]. These measurements clearly demonstrated that dry friction in the single-asperity limit is underdamped and thus the PT model is deemed appropriate to describe many fundamental properties of dry friction.

The present experiment broadens the study of dry friction into the regime of multi-asperity dynamics and so does our model, which provides an intermediate level description bridging the gap between the microscopic behavior of individual slips and the macroscopic laws of solid friction. Because the decay rate that determines how long the inertia effect will last scales as γ/m, which is independent of the system size (or mass), dry friction in the multi-asperity regime will remain underdamped as that in the single-asperity regime. As a result, the predictions of the ABBM model and its variations, which are valid only for the overdamped systems, will not be applicable to dry friction at mesoscale.

In recent years, many attempts have been made to include some new features of the random force field into the PT model, such as adding a Gaussian white noise to the PT model to account for the thermal activation effect [15], assuming $F_i(x)$ results from a fractal potential [29], or assuming $F_i(x)$ to follow the Amontons' law of friction, $F_i(x) = \mu N$, with the frictional coefficient μ following the (empirical) rate (or speed-dependent) and state (or time-dependent) friction law [23, 24]. These models studied the transition boundary between the steady sliding and stick-slip motion, as predicted by the PT model, and provided a more realistic but complex framework for the pinning force field. However, to our knowledge, these modified PT models have not reported any result on the slip length distribution.

When a sharp AFM tip scans over a fractal-like rough surface, as proposed in Ref. [29], the tip can feel the entire roughness landscape of the underlying surface. In this case, the resulting slip lengths may follow a power-law distribution, which is linked to the statistics of the surface roughness or the pinning force field. Because it is solely determined by the self-similar nature of the surface roughness, the power-law distribution in this single-asperity regime does not have any connection with the avalanche dynamics. In this work, we demonstrate that the stick-slip friction in mesoscale is an emergent (or self-organized) phenomenon, which is realized in the multiple-asperity regime. In this case, the power-law distribution of slip length δx_s arises from the spontaneous response of multiple asperities at the contact to the stick-slip instabilities and is not necessarily linked to the self-similar nature of the surface roughness. Instead of assuming the total pinning force $F_i(x)$ in Eq. (14) has a specific fractal form, our model adopts a more general assumption that the total force $F_i(x)$ resulting from the multiple-asperity pinning is Brownian correlated.

In fact, the assumption that $F_i(x)$ is Brownian correlated is a general feature of the random pinning force field. To show this, we rewrite Eq. (9) as

$$F_i(x_s) = \iint_{A(x_s)} [\sigma(x, y; x_s)dy]dx = \int_{L_x} h(x; x_s)dx, \tag{15}$$

where $h(x; x_s) = \int_{L_y} \sigma(x, y; x_s)dy$ is an effective interfacial tension. Because both $\sigma(x, y; x_s)$ and $F_i(x_s)$ are real physical quantities, they must have a finite variance. Consequently, the integrand $h(x; x_s)$ in Eq. (15) will also have a finite variance σ_h^2, which is related to the frictional force variance σ_F^2 by $\sigma_F^2 = \sigma_h^2 L_x$. In fact, this statement is true even for fractal-like rough surfaces, because the self-similar feature of the actual solid surfaces always has two cutoff lengths. The lower cutoff is determined by the smallest asperity size (e.g., the grain size of the sandpaper), whereas the higher cutoff must be bounded by the system size.

The last equality in Eq. (15) states that the total pinning force $F_i(x_s)$ is a running sum of $h(x; x_s)$ along the moving direction. According to the running average (or sum) theorem [30], the auto-correlation function $c(\lambda) = \langle F_i(x_s)F_i(x_s + \lambda)\rangle$ of $F_i(x_s)$ has the form

$$c(\lambda) \simeq \sigma_h^2(L_x - \lambda) = \sigma_F^2 \left(1 - \frac{\lambda}{L_x}\right), \tag{16}$$

where $L_x - \lambda$ is the overlap region between $F_i(x_s)$ and $F_i(x_s + \lambda)$ (for small values of λ/L_x). The mean-squared force-difference $\langle \Delta F_i^2(\lambda)\rangle = \langle |F_i(x_s + \lambda) - F_i(x_s)|^2\rangle$ then

takes the form

$$\langle \Delta F_i^2(\lambda) \rangle = 2\sigma_F^2 - 2c(\lambda) = 2D\lambda, \quad (17)$$

where $D = \sigma_F^2/L_x = \sigma_h^2$.

Differentiating both sides of Eq. (14) with respect to x and using $d/dx = \frac{1}{v}\frac{d}{dt}$, where v ($\equiv dx/dt$) is the velocity of the CoM, we have

$$m\frac{d^2v}{vdt^2} + \gamma\frac{dv}{vdt} = k_0(\frac{U}{v} - 1) + w(x), \quad (18)$$

where $w(x) = dF_i(x)/dx$ has a zero mean and obeys the relation $\langle w(x)w(x')\rangle = 2D\delta(x-x')$. Multiplying Eq. (18) by v, we obtain the equation of the slip velocity $v(t)$,

$$m\frac{d^2v}{dt^2} + \gamma\frac{dv}{dt} = k_0(U - v) + \sqrt{v}\eta(t), \quad (19)$$

where $\eta(t) = \sqrt{v}w$ is a Gaussian white noise, having a zero mean and a correlation $\langle \eta(t)\eta(t')\rangle = 2D\delta(t - t')$. Now making a variable replacement with $v = VU$ and $t = T\sqrt{m/k_0}$, we obtain

$$k_0U\frac{d^2V}{dT^2} + \frac{\gamma U}{\sqrt{m/k_0}}\frac{dV}{dT} = k_0U(1-V) + \sqrt{UV}\xi(T). \quad (20)$$

The new noise $\xi(T)$ has a correlation $\langle \xi(T)\xi(T')\rangle = (2D\sqrt{m/k_0})\delta(T - T')$, which can be obtained by using the correlation function $\langle \eta(t)\eta(t')\rangle = 2D\delta(\sqrt{m/k_0}(T - T')) = (2D\sqrt{k_0/m})\delta(T - T')$ and letting $\eta(t) = \xi(T)$. Simplifying Eq. (20) yields

$$\frac{d^2V}{dT^2} + \gamma'\frac{dV}{dT} = 1 - V + \sqrt{V}\zeta(T), \quad (21)$$

where $\gamma' = \gamma/\sqrt{mk_0}$ and $\langle \zeta(T)\zeta(T')\rangle = 2D'\delta(T - T')$ with $D' = D/\sqrt{mk_0^3U^2}$.

C. Numerical results of the under-damped spring-block model

Equation (21) has two parameters, the normalized damping coefficient γ' and the normalized "diffusion coefficient" D' of the random pinning force field. In the absence of the random force field, i.e., when $D' = 0$, V oscillates and converges to 1 for $\gamma' \leq 2$, indicating that the system is in the under-damped regime. For $\gamma' > 2$, V monotonically decays to 1, suggesting the system is over-damped. On the other hand, when $D' \gg 1$, Eq. (21) suggests that the motion of the CoM is dominated by the pinning-depinning dynamics in both the under-damped and over-damped regimes.

Equation (21) can be solved numerically using the Euler-Maruyama method, following the protocol adopted in Ref. [26]. A slip starts at a zero acceleration ($\frac{dV}{dT}(T = 0) = 0$) and a very small positive initial velocity $V_0 \ll 1$ and stops when V first becomes negative after a period T_s. Since the velocity should always be positive, a new slip starts on the same initial condition but with a different realization of the noise. The total displacement during a slip is defined as the slip length, $\delta x_s \equiv \int_0^{T_s} VdT$. The initial velocity V_0 used here only affects the lower cut-off of the power-law regime of the slip-size distribution $P(\delta x_s)$. It is found that a smaller value of V_0 gives rise to a smaller value of the lower cut-off. We chose $V_0 = 10^{-4}$ in this work in order to have a large power-law range.

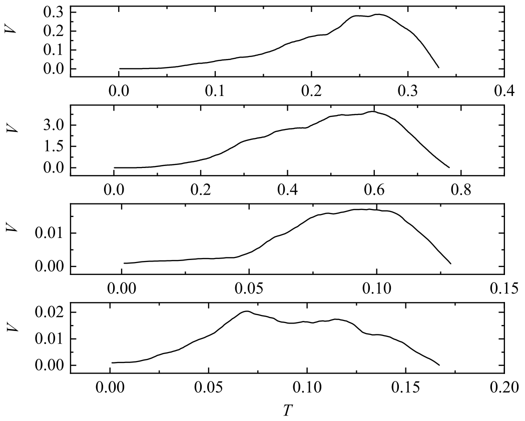

Supplementary Fig. 20. Examples of the velocity trajectory during a slip. The four velocity time series $V(T)$ are obtained from the numerical simulation of Eq. (21) with $\gamma' = 2$ and $D' = 500$.

Supplementary Fig. 20 shows four examples of the velocity time series $V(T)$ during a slip, which are obtained at $\gamma' = 2$ and $D' = 500$. It is seen that these slip events have different slip durations T_s and different maximum velocities. The velocity trajectories show an overall acceleration phase followed by a deceleration phase, but the actual shape of $V(T)$ varies for different slips, which is indeed a manifestation of the stochastic nature of avalanches. By carefully examining the individual velocity trajectories, we find that neither the velocity $V(T)$ nor the slip duration T_s remain constant during the slips.

Supplementary Fig. 21 show the PDFs $P(\delta x_s)$ of the numerically calculated slip length δx_s for different values of D', when the system is in the under-damped regime with $\gamma' = 1$. It is seen that in the strong pinning limit $D' \gg 1$ (e.g., $D' = 500$), the obtained $P(\delta x_s)$ follows a power-law distribution, $P(\delta x_s) \sim \delta x_s^{-\epsilon}$, over a wide range of δx_s for seven decades. Note that the power-law distribution of slip length δx_s arises from the dynamics of stick-slip instabilities and is not necessarily linked to a specific feature of the surface roughness. There is only a general requirement for the rough surface under study that the corresponding pinning force field $F_i(x)$ is random enough and Brownian-correlated. Therefore, the stick-slip friction in mesoscale is an emergent (or self-organized) phenomenon, which is realized in the multiple-asperity regime.

As D' decreases, the power-law range is reduced mainly

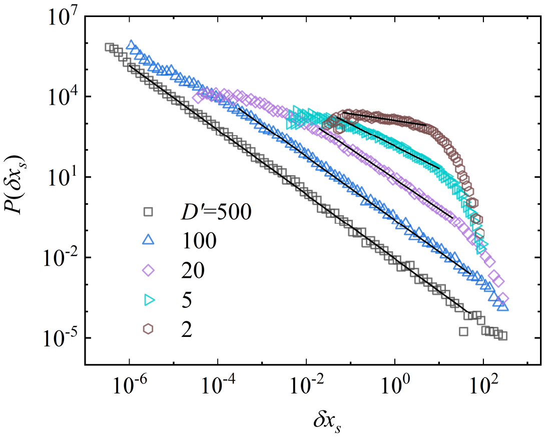

Supplementary Fig. 21. **Numerical results of the distribution of slip length.** The PDFs of δx_s are obtained for different values of D' at $\gamma' = 1$. For clarity, the colored curves (except the black squares) are shifted vertically. The solid lines indicate the power-law fits, $P(\delta x_s) \sim \delta x_s^{-\epsilon}$, to each set of data in the power-law region. The fitted values of ϵ are, respectively, 1.20 ($D' = 500$), 1.18 ($D' = 100$), 1.11 ($D' = 20$), 0.83 ($D' = 5$) and 0.25 ($D' = 2$).

due to the increase of the lower cutoff of the slip length. At the same tine, the exponent ϵ also decreases. The actual value of ϵ in Supplementary Fig. 21 is determined from a compensated plot of $P(\delta x_s)\delta x_s^{\epsilon}$ as a function of δx_s. The value of ϵ is adjusted so that it gives the largest plateau region, in which we have $P(\delta x_s)\delta x_s^{\epsilon} =$ constant. The error bar of the final value of ϵ is determined by the fitting uncertainty of ϵ. This method of using compensated plots to determine the power-law exponent is widely used in the study of the scaling properties of turbulent flows [31]. It is also found that for $D' \ll 1$, the motion of the CoM shows quasi-periodic oscillations, as expected for the steady state motion of the CoM when the pinning effect becomes negligible.

The experimentally obtained power-law exponent $\tau = 0.72$ for the 2D probe is smaller than the numerically predicted value of $\epsilon = 1.2$. One possible reason is that the slip length δx_s measured in the experiment is actually bounded by the physical dimensions of the apparent contact area. Because its length along the scanning direction is severely suppressed, the quasi-1D probe will detect more "line-shaped" (or quasi-1D) slips along the lateral direction. On the other hand, the 2D probe has a square shape and will detect more 2D slips at the interface. As Eq. (14) uses a 1D pinning force field to describe the stick-slip friction of a "contact line", it is expected to work better for quasi-1D (or "line-shaped") slips. Further investigation is needed in order to include such effects as the 2D pinning force field, the geometry and dimensionality of the scanning probe, spatial averaging and finite size into the theoretical model.

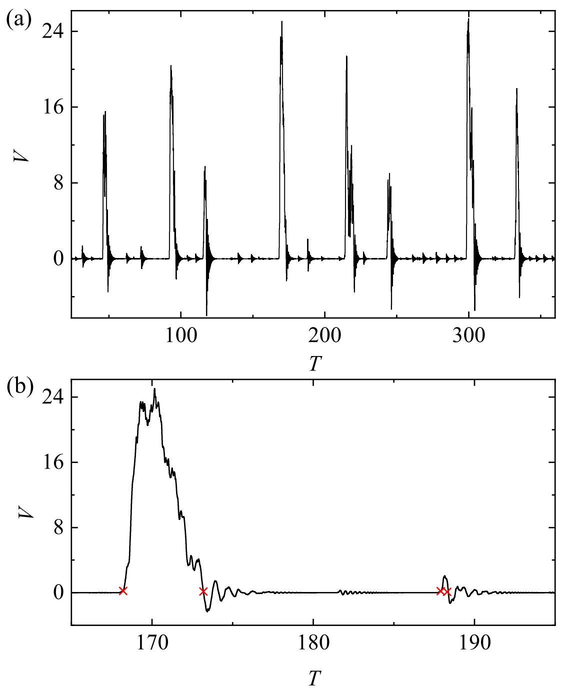

Supplementary Fig. 22. **A steady-state solution of the equation of motion for the underdamped spring block model.** (a) An example of the steady-state velocity time series $V(T)$ obtained by integrating Eq. (14) for a given realization of the Brownian-correlated pinning force field $F_i(x)$. The dimensionless parameters used here are $\gamma' = 2$ and $D' = 500$. (b) A magnified view of $V(T)$ during the period of $T = 165$–195. The two pairs of red crosses indicate the beginning and ending of the two slip events.

D. Steady-state velocity time series of the underdamped spring block model

Another way to obtain the distribution of slip lengths is to directly integrate Eq. (14) for a given realization of the pinning field $F_i(x)$ that is Brownian-correlated. By using the dimensionless time variable $T = t/\sqrt{m/k_0}$ and space variable $X = x/(U\sqrt{m/k_0})$, Eq. (14) can be converted to a dimensionless form with two dimensionless control parameters. The first one is the effective damping coefficient $\gamma' = \gamma/\sqrt{mk_0}$ and the other is the effective fluctuation magnitude $D' = D/(mk_0U^2)$ of the pinning force filed. Supplementary Fig. 22(a) shows a steady-state velocity time series $V(T)$ obtained at $\gamma' = 2$ and $D' = 500$. It is seen that $V(T)$ exhibits alternating quiet periods (stick state) and burst periods (slip state), indicating the stick-slip dynamics.

Supplementary Fig. 22(b) shows a magnified view of $V(T)$ during the period of $T = 165$–195, where two slip events are observed and marked by two pairs of red crosses to indicate their start and end times. It is seen that the large velocity burst is followed by a decaying oscillation. This oscillation is caused by the under-damped nature of Eq. (14). Because the pinning force field is ran-

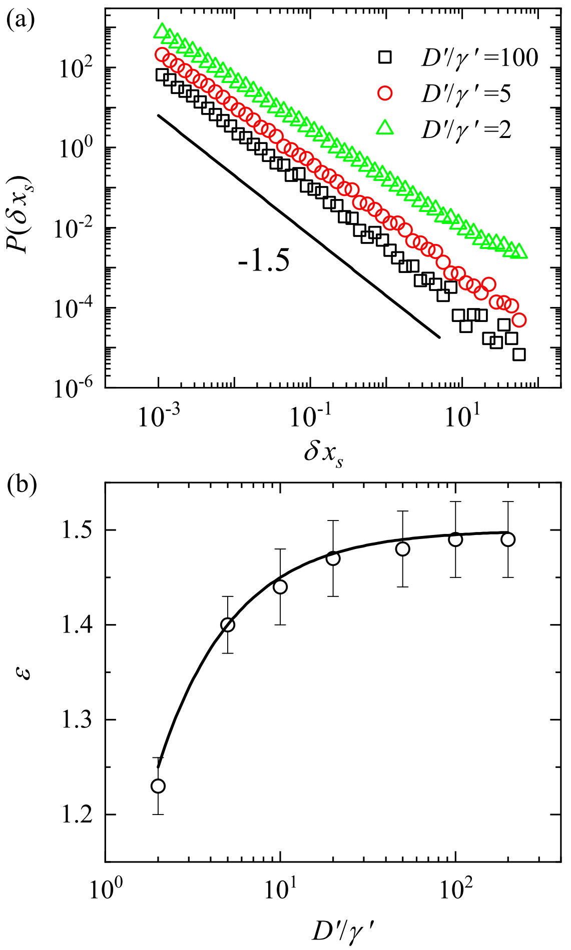

Supplementary Fig. 23. **Numerical results in the over-damped regime.** (a) Calculated PDF $P(\delta x_s)$ of slip length δx_s for different values of D'/γ' in the over-damped regime with $\gamma' = 100$. The solid line indicates a power law with an exponent $\epsilon = -1.5$. (b) Obtained power-law exponent ϵ as a function of D'/γ' in the over-damped regime with $\gamma' = 100$. The black solid line shows the theoretical prediction of the ABBM model, $\epsilon = \frac{3}{2} - \frac{1/2}{(D'/\gamma')}$. The error bars show the standard deviation of the fitted power-law exponent ϵ.

dom, the shape of the oscillation is irregular and cannot be modelled by a well-defined function, such as that for the simple damped oscillation in the absence of the random pinning force field. It is thus very difficult to set up a uniform threshold value to distinguish the "after-slip" oscillations from the actual small slip events in the time series of the steady-state velocity. Our numerical protocol, as described in Sec. III.C, effectively avoids this problem.

Supplementary Fig. 22 also reveals that in the strong pinning or low speed limit (i.e., at the large value of $D' = 500$), the stick period between the neighboring large bursts (slips) is sufficiently long such that the system has enough time for the "after-slip" oscillations to die out. In this case, removing the small "after-slip" oscillations with our numerical protocol does not affect the statistical properties of the large burst (slip) events. It allows us to filter out unwanted noise and focus on the main effect of the large slip events, which are generated by the under-damped dynamics as well.

E. Numerical results of the over-damped spring-block model

When $m = 0$ in Eq. (14) or $\gamma' \gg 1$ in Eq. (21), our spring-block model effectively reduces to the ABBM model for the over-damped systems. Therefore, one can study the avalanche dynamics in the over-damped systems using Eq. (21) with a large value of γ' (without actually setting $m = 0$). In this case, Eq. (21) has only one dimensionless control parameter, namely, D'/γ'. Supplementary Fig. 23(a) shows the calculated PDFs $P(\delta x_s)$ of the slip length δx_s for different values of D'/γ' in the over-damped regime with $\gamma' = 100$. It is found that all the PDFs $P(\delta x_s)$ can be well-described by a power-law distribution, $P(\delta x_s) \sim \delta x_s^{-\epsilon}$. The obtained power-law exponent ϵ increases with D'/γ' for small values of D'/γ'. In the strong pinning limit ($D'/\gamma' = 100$), the obtained exponent ϵ reaches an asymptotic value $\epsilon \simeq 1.5$ (solid line), which agrees well with the prediction of the ABBM model.

Supplementary Fig. 23(b) shows how the obtained power-law exponent ϵ changes with D'/γ' over a wide range of D'/γ'. It is seen that our numerical results agree well with the prediction of the ABBM model, $\epsilon = \frac{3}{2} - \frac{1/2}{(D'/\gamma')}$ (solid line), which has been derived both analytically and numerically in the previous studies [25, 26]. Supplementary Fig. 23(b) thus demonstrates that our numerical protocol of simulating the slip events works well and that the avalanche dynamics in the under-damped regime is very different from that in the over-damped regime.

Supplementary References

[1] Gujrati, A., Khanal, S. R., Pastewka, L., & Jacobs, T. D. B. Combining TEM, AFM, and profilometry for quantitative topography characterization across all scales. *ACS Appl. Mater. Interfaces* **10**, 29169-29178 (2018).

[2] Jacobs, T. D. B., Junge, T., & Pastewka, L. Quantitative characterization of surface topography using spectral analysis. *Surf. Topogr.: Metrol. Prop.* **5**, 013001 (2017).

[3] Gujrati, A., Sanner, A., Khanal, S. R., Moldovan, N., Zeng, H., Pastewka, L., & Jacobs, T. D. B. Comprehensive topography characterization of polycrystalline diamond coatings. *Surf. Topogr.: Metrol. Prop.* **9**, 014003 (2021).

[4] Hutter, J. L., & Bechhoefer, J. Calibration of atomic-force microscope tips. *Rev. Sci. Instrum.* **64**, 1868 (1993).

[5] Xiong, X. M., Guo, S., Xu, Z. L., Sheng, P., & Tong, P. Development of an atomic-force-microscope-based hanging-fiber rheometer for interfacial microrheology. *Phys. Rev. E* **80**, 061604 (2009).

[6] Cannara, R. J., Eglin, M., & Carpick, R. W. Lateral force calibration in atomic force microscopy: A new lat-

eral force calibration method and general guidelines for optimization. *Rev. Sci. Instrum.* **77**, 053701 (2006).

[7] Xie, H., Vitard, J., Haliyo, S., Régnier, S., & Boukallel, M. Calibration of lateral force measurements in atomic force microscopy with a piezoresistive force sensor. *Rev. Sci. Instrum.* **79**, 033708 (2008).

[8] Liu, E., Blanpain, B., & Celis, J. P. Calibration procedures for frictional measurements with a lateral force microscope. *Wear* **192**, 141-150 (1996).

[9] Morel, N., Ramonda, M., & Tordjeman, Ph. Cantilever calibration for nanofriction experiments with atomic force microscope. *Appl. Phys. Lett.* **86**, 163103 (2005).

[10] Hopcroft, M. A., Nix, W. D., & Kenny, T. W. What is the Young's modulus of silicon? *J. Microelectromech. Syst.* **19**, 2 (2010).

[11] Mate, C. M. *Tribology on the Small Scale: A Bottom Up Approach to Friction, Lubrication, and Wear* (Oxford University Press, Oxford, 2008).

[12] Leadbetter, M. R., Lindgren, G., & Rootzén, H. *Extremes and Related Properties of Random Sequences and Processes.* p. 267 (Springer, New York, 2004).

[13] Greenwood, J. A., & Williamson, J. B. P. Contact of Nominally Flat Surfaces. *Proc. R. Soc. Lond. A* **295**, 300-319 (1966).

[14] Medyanik, S. N., Liu, W. K., Sung, I. H., & Carpick, R. W. Predictions and observations of multiple slip modes in atomic-scale friction. *Phys. Rev. Lett.* **97**, 136106 (2006).

[15] Heslot, F., Baumberger, T., Perrin, B., Caroli, B., & Caroli, C. Creep, stick-slip, and dry-friction dynamics. *Phys. Rev. E* **49**, 4973 (1994).

[16] Socoliuc, A., Bennewitz, R., Gnecco, E., & Meyer, E. Transition from stick-slip to continuous sliding in atomic friction: entering a new regime of ultralow rriction. *Phys. Rev. Lett.* **92**, 13 (2004).

[17] Zwörner, O., Hölscher, H., Schwarz, U. D., & Wiesendanger, R. The velocity dependence of frictional forces in point-contact friction. *Appl. Phys. A* **66**, S263-S267 (1998).

[18] Vanossi, A., Manini, N., Urbakh, M., Zapperi, S., & Tosatti, E. Modeling friction: From nanoscale to mesoscale. *Rev. Mod. Phys.* **85**, 529-552 (2013).

[19] Helman, J. S., Baltensperger, W., & Holyst, J. A. Simple model for dry friction. *Phys. Rev. B* **49**, 3831 (1994).

[20] Li, Q., Dong, Y., Perez, D., Martini, A., & Carpick, R. W. Speed dependence of atomic stick-slip friction in optimally matched experiments and molecular dynamics simulations. *Phys. Rev. Lett.* **106**, 126101 (2011).

[21] Gnecco, E., Bennewitz, R., Gyalog, T., Loppacher, Ch., Bammerlin, M., Meyer, E., & Güntherodt, H.-J. Velocity dependence of atomic friction. *Phys. Rev. Lett.* **84**, 1172 (2000).

[22] Popov, V. L., & Gray, J. A. T. Prandtl-Tomlinson model: History and applications in friction, plasticity, and nanotechnologies. *Z. Angew. Math. Mech.* **92**, 9 (2012).

[23] Gu, J. C., Rice, J. R., Ruina, A. L., & Tse, S. T. Slip Motion and stability of a single degree of freedom elastic system with rate and state dependent friction. *J. Mech. Phys. Solids* **32**, 167-196 (1984).

[24] Shroff, S. S., & de Boer, M. P. Full assessment of micromachine friction within the rate-state framework: Experiments. *Tribol. Lett.* **63**, 31 (2016).

[25] Colaiori, F. Exactly solvable model of avalanche dynamics for Barkhausen crackling noise. *Adv. Phys.* **57**, 4 (2008).

[26] LeBlanc, M., Angheluta, L., Dahmen, K., & Goldenfeld, N. Universal fluctuations and extreme statistics of avalanches near the depinning transition. *Phys. Rev. E* **87**, 022126 (2013).

[27] Müser, M. H., Urbakh, M., & Robbins, M. O. Statistical Mechanics of Static and Low-velocity Kinetic Friction. *Adv. Chem. Phys.* **126**, 187 (2003).

[28] Klein, J. Frictional Dissipation in Stick-Slip Sliding. *Phys. Rev. Lett.* **98**, 056101 (2007).

[29] Filippov, A. E., & Popov, V. L. Fractal Tomlinson model for mesoscopic friction: From microscopic velocity-dependent damping to macroscopic Coulomb friction. *Phys. Rev. E* **75**, 027103 (2007).

[30] Anderson, O. D. Moving average processes. *J. R. Stat. Soc. Series D: The Statistician*, **24**, 4 (1975).

[31] Ahlers, G., Grossmann, S., & Lohse, D. Heat transfer and large-scale dynamics in turbulent Rayleigh-Bénard convection. *Rev. Mod. Phys.* **81**, 503 (2009).
